# Survival of ampicillin-treated uropathogenic *Escherichia coli* is independent of single-cell growth rates

**DOI:** 10.1038/s44259-025-00180-6

**Published:** 2026-02-02

**Authors:** Yoshiko Miyahara, François Signorino-Gelo, Nicolas Elian Michel Lonchampt, Paul Murima, John D. McKinney, Neeraj Dhar

**Affiliations:** 1 Global Health Institute, School of Life Sciences, Swiss Federal Institute of Technology in Lausanne (EPFL), Lausanne, Switzerland; 2 Safety Science Research Laboratories, Kao Corporation, Tochigi, Japan; 3 Vaccine and Infectious Disease Organization (VIDO), University of Saskatchewan, Saskatoon, SK Canada; 4 Department of Biochemistry, Microbiology and Immunology, College of Medicine, University of Saskatchewan, Saskatoon, SK Canada; 5 Vaccinology & Immunotherapeutics Program, School of Public Health, University of Saskatchewan, Saskatoon, SK Canada

**Keywords:** Diseases, Microbiology

## Abstract

The refractoriness of persistent infections to antibiotics necessitates lengthy treatment regimens to prevent therapeutic failures and relapses. Persistence has been attributed to entry of a small fraction of bacterial cells into a slowly growing or non-growing physiological state, which is thought to protect them against antibiotics targeting growth-related processes. However, these conclusions are largely based on studies conducted with lab-adapted strains carrying mutations that confer abnormally high levels of persistence. Here, we perform single-cell studies of ampicillin-mediated killing and persistence in a clinical isolate of uropathogenic *Escherichia coli* (UPEC). We show that the majority of surviving cells are growing and dividing normally at the time of ampicillin exposure. Conversely, we find that the majority of non-growing cells are readily killed by ampicillin exposure. These findings challenge the widespread assumption that bacterial dormancy and persistence are inextricably linked.

## Introduction

Antibiotic resistance is predicted to be the next global pandemic and is estimated to be responsible for more than 10 million deaths by 2050^1^. Recent studies have highlighted that persistence of bacteria against killing by antibiotics enables the survivors to become resistant^2–5^. Bacterial persistence is a phenotypic state in which a small subpopulation of cells in an isogenic population can survive killing by antibiotics. Bacterial persistence is quite universal and has been documented in different species and against wide range of antibiotics. Because bacterial persistence is a multifactorial phenomenon and occurs more frequently, it provides a background for the emergence of resistance. As a result, incomplete or “fractional” killing of bacterial populations by antibiotics is believed to play an important role in therapeutic failures and relapses after treatment^6–8^.

Fractional killing was first described by Bigger^9^, who hypothesized that small numbers of bacteria might escape killing by entering a reversible state of dormancy. This interpretation seemed logical because antibiotics target processes involved in cell growth and division, such as cell wall biogenesis, which may be less important for survival in dormant cells. During infection, host defenses restrict nutrient availability to bacteria, inhibiting proliferation^10–13^. This inactivation of antibiotic targets caused by starvation-induced growth arrest is thought to be a survival strategy under drug exposure^14^. However, direct evidence supporting Bigger’s hypothesis was not obtained until much later, when time-lapse imaging revealed that ampicillin-refractory “persisters” comprise a subpopulation of slowly growing or non-growing cells^15^. Although compelling, this work was conducted using high-persistence (*hip*) mutants of a lab-adapted strain of *Escherichia coli* (MG1655), and it remains unclear to what extent the authors’ conclusions can be generalized to other bacteria. Recent studies have indicated that bacterial dormancy might not be a universal explanation for fractional killing by antibiotics^16–21^, although it has also been suggested that these may be atypical cases^6,7^.

In the present study, we decided to directly address the role of growth rate on antibiotic persistence by employing a uropathogenic strain of *Escherichia coli* (UPEC), CFT073. UPEC is responsible for about 75% of urinary tract infections (UTIs), which affects about 150 million people worldwide and is the second most common cause for prescription of antibiotics^22,23^. UTIs are also characterized by high rates of recurrence and persistence. Using microfluidic devices and time-lapse microscopy, we studied how growth rates affect persistence of UPEC against ampicillin. In nutrient-rich media, survival was independent of growth rate, aligning with our findings in mycobacteria^17^ but differing from observations in lab strains of *E. coli*^15^. By modulating growth using minimal media with non-metabolizable glucose, we show that survival did not require bacteria to be in a state of growth arrest, and single-cell analysis revealed no correlation between death rate and growth rate. Both growing and non-growing cells could survive ampicillin, and neither RpoS nor TolC contributed to survival. These results underscore distinct survival mechanisms in pathogenic *E. coli* and demonstrate the value of single-cell approaches.

## Results

**Fig. 1:**
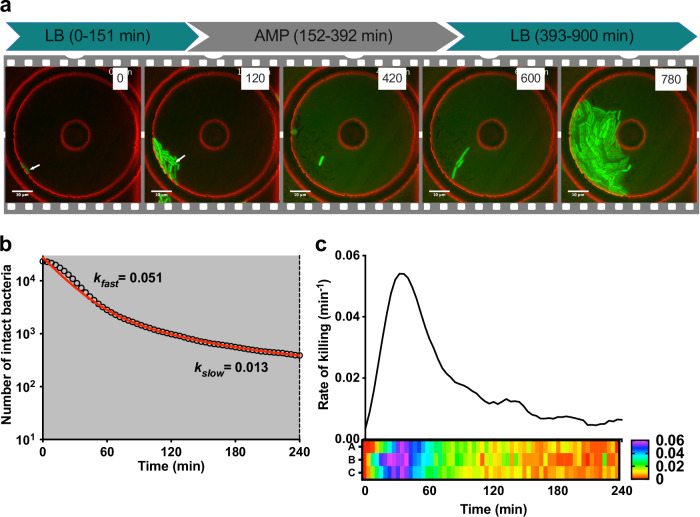
UPEC persistence during ampicillin exposure. **a** A representative series of time-lapse images of ampicillin-treated UPEC strain CFT073 expressing YFP. The fluorescence (green) and phase (red) images are merged. The large outer circle is the SU-8 microchamber patterned on the coverslip and the small inner circle is an SU-8 pillar to support the overlying membrane. Scale bar, 10 μm. Time (minutes) is indicated on the top right. Ampicillin (50 μg m^−^^1^) was added to the flow medium at 152–392 min. In this example, one cell survives antibiotic exposure (white arrow) and repopulates the chamber after ampicillin withdrawal. **b**, **c** Surviving UPEC cells were enumerated by time-lapse microscopy during exposure to 50 μg ml^−1^ ampicillin. Data from three independent experiments (23,350 cells). **b** Lysis kinetics over time of ampicillin-treated UPEC. The red line depicts the two-phase fitting of cell numbers over time to determine the *k*_*fast*_ and *k*_*slow*_ lysis rates. **c** Upper panel: Change in the rate of lysis of ampicillin-treated UPEC over time. The solid line depicts the average lysis rate smoothed over 40 min. Lower panel: Data from three independent experiments displaying the change in the rate of lysis as a heat map.

To address the role of bacterial growth rate in antibiotic persistence, we investigated ampicillin persistence in UPEC strain CFT073, originally isolated from a patient with acute pyelonephritis^24^. To facilitate direct comparisons with earlier pioneering studies on antibiotic persistence in *E. coli*, which predominantly used β-lactams, we also selected ampicillin as the focus of our study^9,15,25^. We found that the minimum inhibitory concentration (MIC) of ampicillin was comparable between CFT073 (6.5 ± 1.6 μg ml^−1^) and MG1655 (6.2 ± 1.7 μg ml^−1^), an extensively studied non-pathogenic strain of *E. coli* (*P* > 0.05, Welch’s *t*-test). Time-kill experiments using exponentially growing cultures revealed that CFT073 survived at similar frequency as MG1655 when exposed to 50 μg ml^−1^ ampicillin (Fig. S1a). The biphasic nature and fraction of survivors was dose-independent (Fig. S1b). Previous single-cell studies of persistence employed high persistence (*hip*) mutants of MG1655 due to the very low frequency of persistence in the parental strain (about 10^−5^ to 10^−6^)^15^. Although these studies provided compelling evidence for a link between persistence and dormancy at the single-cell level, the use of *hip* mutants raises the question whether this link is also true in wild-type cells. We addressed this question using a derivative of CFT073 expressing yellow fluorescent protein (YFP) to facilitate imaging (Fig. S1c). Bacteria were taken from exponentially growing cultures and seeded onto coverslips that were micropatterned with small microchambers^26^. A layer of cellulose membrane sandwiched between the coverslip and the PDMS device allows diffusion of growth media or antibiotic containing media. The PDMS device has channels for continuous perfusion of the growth media. The microchambers facilitated the growth and division of *E. coli* cells while spatially confining them within individual wells, thereby preventing their loss during media exchange and enabling high-resolution lineage tracking. In a typical experiment, we imaged about 40 microchambers, acquiring images every 4–5 min. CFT073 bacteria were cultured in the microfluidic setup in LB media over a period of at least 2 h. We found that microfluidic cultures of CFT073 in LB medium grew with similar kinetics as conventional batch cultures (specific growth rate, *k* = 0.023 min^−1^; doubling time, *T*_*d*_ = 29.7 min) and was similar to the rates reported for MG1655 (*T*_*d*_ = 26.5 min)^21^. Following addition of 50 μg ml^−1^ ampicillin to the flow medium, cells initially continued to grow and divide before undergoing abrupt loss of cellular fluorescence due to lysis (Fig. 1a; Movies S1–S5). At the single-cell level, CFT073 displayed biphasic kinetics of cell lysis similar to bulk assays of viable colony-forming units (CFU) (Fig. S1a). Fitting the lysis curve with a two-phase decay model yielded lysis rates of 0.051 min^−1^ during the early (lysis) phase and 0.013 min^−1^ during the late (persistence) phase (Fig. 1b). Initially, the single-cell lysis rate increased sharply, peaking around 40 min post exposure, then decreased to a relatively stable rate beginning around 120 min (Fig. 1c). The overall extent of ampicillin-induced cell lysis observed in the microfluidic device was lower than the reduction in CFUs measured in time-kill assays conducted on bulk cultures (Fig. S1a). This observation aligns with recent findings indicating that CFU-based assays can be misleading due to substantial post-exposure killing, whereas real-time single-cell imaging offers a more accurate assessment of antibiotic activity^11^.

**Fig. 2:**
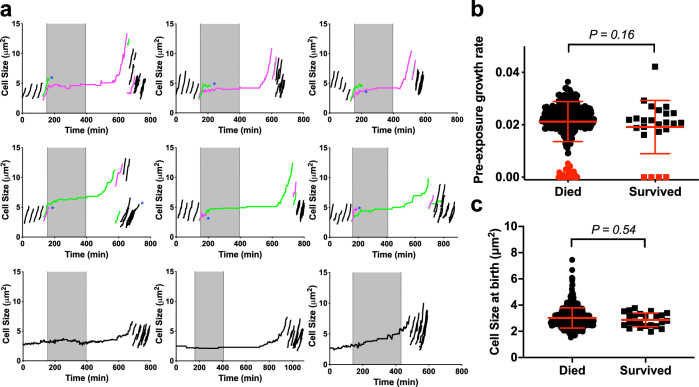
UPEC persistence is not linked to single-cell growth rates. **a** Line-plots depicting cell growth and division in persistent lineages of UPEC strain CFT073. Individual cells were tracked by time-lapse microscopy before, during (grey shading), and after a 4-h exposure to 50 μg ml^−1^ ampicillin. Examples are shown of persistent lineages arising from growing progenitors (top and middle rows) or non-growing progenitors (bottom row). Magenta lines, old-pole cells. Green lines, new-pole cells. Black lines, generations before and after ampicillin treatment. Line-plots of the remaining 15 persistent cell lineages are shown in Fig. S2. **b** Comparison of the pre-ampicillin growth rates (min^−1^) of surviving (squares, *n* = 24) and extinguished (circles, *n* = 351) cell lineages. Slowly growing and non-growing cells (*T*_*d*_>120 min) are shaded in red. Red lines indicate means ± SD. *P* = 0.16 Mann–Whitney *t*-test. **c** Comparison of the cell sizes at birth of bacteria that died (circles, *n* = 428) or survived (squares, *n* = 24) upon exposure to 50 μg ml^−1^ ampicillin. Red lines indicate means ± SD. *P* = 0.54, Mann–Whitney *t*-test.

We investigated the widely accepted idea that “persisters” comprise a subpopulation of slowly growing or non-growing cells^6–8^ by comparing the pre-exposure single-cell growth rates of CFT073 cells that survived or died during ampicillin exposure (Fig. 2a, b). Contrary to expectations, we found that the majority (20/24) of cells that survived and regrew after 4 h of ampicillin treatment were growing and dividing normally prior to ampicillin exposure (Figs. 2b and S2; Movies S1–S4). We did not observe any bias for pole-age amongst the survivors as 11 of the growing survivors were derived from the old-pole sibling, while the remaining 9 were from the new-pole sibling. A minority (4/24) of survivors were not growing at the time when ampicillin was added to the flow medium, but all these cells reinitiated growth after ampicillin withdrawal (Movies S3). In all cases, the progeny of survivors were not resistant to ampicillin, because they lysed when ampicillin was added to the flow medium a second time (Movie S5). Surviving cells did not exhibit any significant differences in birth cell size compared to non-surviving cells (Fig. 2c). At the time of antibiotic washout, surviving cells were about 1.5-fold (range: 1.3–2.2) larger than the average size of unperturbed cells. Following washout, these survivors continued to elongate substantially, increasing in size by an additional ~1.7–3X fold before initiating division. This pronounced elongation was accompanied by extended lag phases, with median lag times of 218 min for growing survivors (*N* = 20) and 290 min for non-growing survivors (*N* = 4).

Surprisingly, we found that the majority (12/16) of non-growing cells and all (17/17) slowly growing cells with a doubling time greater than 120 min were lysed by ampicillin or did not recover after ampicillin withdrawal (Figs. 2b and S2; Movie S4). Furthermore, the pre-ampicillin ensemble growth rates of cells that died (*k* = 0.019 min^−1^) and cells that survived (*k* = 0.020 min^−1^) were not significantly different (*P* = 0.16, Mann–Whitney *t* test) (Fig. 2b). These observations held true when the period of ampicillin exposure was extended from 4 to 6 h (Fig. S3). We conclude that entry into a slowly growing or non-growing state is neither necessary nor sufficient for bacterial survival during ampicillin exposure. We observed similar results with the enterohemorrhagic *E. coli* (EHEC) strain CIP 105917 (Fig. S4, and Movie S16), wherein we detected four bacteria (amongst 7801 imaged) that survived ampicillin treatment of which three were growing and dividing normally prior to antibiotic exposure.

### αMG influences growth and killing kinetics in batch cultures

To modulate the growth rate of UPEC as a function of nutrient availability, we cultured CFT073 in M9 minimal medium supplemented with a single carbon source (0.2% glucose). To alter the growth rate without inducing pleiotropic responses to starvation, we added α-methylglucoside (αMG), a non-metabolizable glucose analog, at a concentration 40X higher than that of glucose. Initially, we tested how the growth of CFT073 and the killing dynamics upon exposure to ampicillin are influenced by αMG at the population level. As expected, the growth rate was significantly slowed when M9 containing 40X αMG was used to culture bacteria, M9: *k* = 0.015 min^−1^, doubling time, *T*_*d*_ = 46.5 min; M9+40X αMG: *k* = 0.0023 min^−1^, doubling time, *T*_*d*_ = 292.3 min (Fig. S5a). CFT073 bacteria were cultured under these conditions, and cells from exponential phase were exposed to 50 μg ml^−1^ ampicillin and the fraction of surviving CFU was enumerated. CFT073 exhibited persistence against ampicillin, characterized by biphasic kill-kinetics under these conditions of slowed growth (Fig. S5b). Supplementation of 40X αMG resulted in enhancement of persistence levels (250 to 700-fold) after 4 to 8-h exposure of ampicillin. These results would suggest that growth rate influences the killing kinetics and persistence levels of CFT073 against ampicillin at the population level, which is consistent with what was reported for lab strains of *E. coli*^14,21,27,28^.

### Growth modulation influences bacterial survival against ampicillin in microfluidic devices

**Fig. 3:**
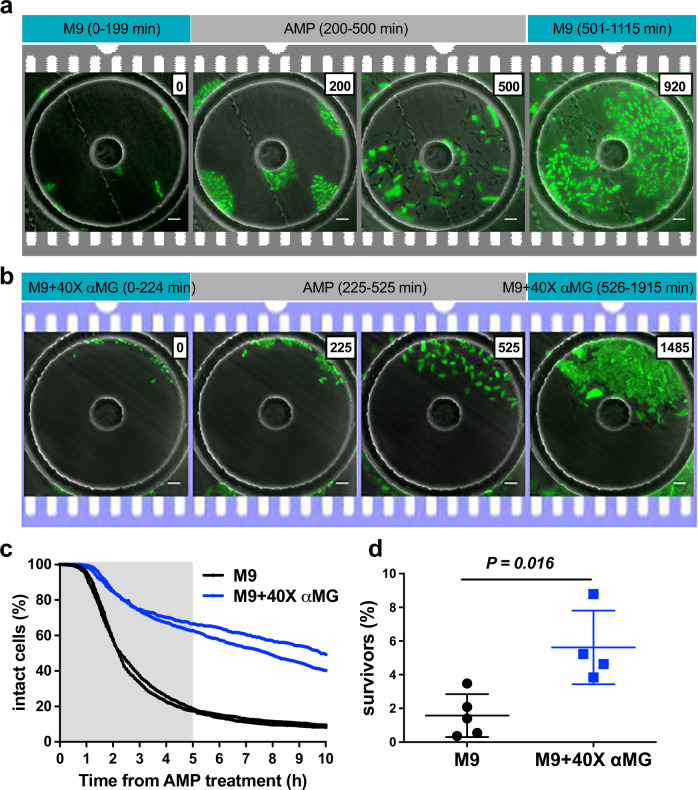
Ampicillin-mediated lysis is influenced by αMG in microfluidic cultures. Representative image series in M9 (**a**) and in M9+40X αMG (**b**). CFT073 expressing YFP were imaged on fluorescence and phase channels at 5 min intervals and exposed to ampicillin (AMP) from 200 to 500 min and from 225 to 525 min in M9 and in M9+40X αMG, respectively. This experiment was repeated more than 4 times in each medium. Scale bar, 5 μm. **c** Total percentage of intact cells during and after ampicillin treatment when CFT073 were cultured in M9 (black) or in M9+40X αMG (blue). Number of analyzed cells at the beginning of these experiments were 3461 and 2004 in M9 and in M9+40X αMG, respectively. AMP treatment is indicated by grey shading. Representative duplicates are shown. **d** Frequency of persisters in M9 (black) and in M9+40X αMG (blue). Means ± SD (*n* ≥ 4) are shown. *P* = 0.016, Mann–Whitney test.

As previously described, we employed our microfluidic culture system combined with time-lapse microscopy to assess the role of modulated growth rates on ampicillin persistence at the single-cell level. Cells cultured in M9 medium or in M9+40X αMG to mid-exponential phase, were seeded into the microfluidic device and grown in the corresponding medium without antibiotic for 3 h. Subsequently, they were exposed to 50 μg ml^−1^ ampicillin for a period of 5 h, followed by washout of the compound (Fig. 3a, b). Cell death was quantified by loss of YFP fluorescence following cell lysis, and survivors were identified upon their resumption of growth and division after the removal of ampicillin. Consistent with our observations in batch cultures, the inclusion of αMG in growth media slowed ampicillin-mediated lysis (Fig. 3c). While the majority of cells lysed during antibiotic exposure, some cells lysed after withdrawal of ampicillin (Fig. 3c). During the course of ampicillin exposure and the subsequent recovery process, cells exhibited heterogeneous morphological changes, including elongation, membrane blebbing, and transformations into L-form or amoeboid-like shapes (Movies S6 and S7) - patterns consistent with previous reports involving other antibiotics and bacterial species^19,21,29–31^. Although cells cultured in M9+40X αMG condition had smaller sizes, ampicillin-induced morphological changes occurred irrespective of the growth conditions. Also, there was no direct correlation between the changes in cell morphology and cell survival; some cells were able to regrow without undergoing significant morphological changes, while others managed to regrow after fragmenting from an amoeboid L-form shape. In the latter case, the cells initially exhibited irregular shapes during the early stages of regrowth but gradually returned to normal cell morphology as division progressed. Additionally, we observed cells that underwent similar morphological changes but lysed during the recovery process. As seen in batch cultures, the frequency of survivors was higher in the presence of αMG (Fig. 3d). Similar to observations in LB medium, CFT073 cultured in M9 media exhibited a markedly higher survival rate in microfluidic cultures (5-h exposure: 1.6 ± 1.3%), with a 69-fold increase compared to batch cultures under the same conditions (5-h exposure: 0.02 ± 0.02%). A significant disparity between these two modes of culturing is the way in which the cells are collected upon antibiotic washout. In batch cultures, the surviving cells are centrifuged, washed and plated on solid media (LB-agar) whereas in the microfluidic device cultures, the antibiotic is washed out in a continuous flow-setup and the surviving cells are allowed to regrow in liquid medium (M9 or M9+40X αMG) flowing through the device. This difference raises the possibility that a large fraction of viable cells in batch cultures are getting killed during the plating procedure or the recovery process on solid media after drug washout. Moreover, shift from nutrient-poor M9-based medium to nutrient-rich LB agar medium can also influence the survival frequency^11,32^. To test this possibility, we enumerated bacterial counts in batch cultures using the Most Probable Number (MPN) assay using the same medium for recovery as was used during antibiotic treatments^33^. This assay is gentler and allows recovery of bacteria in liquid medium instead of solid medium and therefore is more like the microfluidic-device culture method. Indeed, ~50-fold more UPEC survivors were recovered from M9 batch cultures when survivor counts were determined using the MPN assay (Fig. S5c). The fraction of survivors measured by MPN assay (1.1 ± 1.7%) were more similar to those obtained in microfluidic-device cultures than those measured by solid media-CFU count assay. It is possible that the cells recovering on liquid medium includes an antibiotic-injured sub-population such as amoeboid L-form bacteria that might be sensitive to the plating procedure and nutrient-shift. Interestingly, when M9 was supplemented with αMG, the fraction of survivors was similar in both batch (6-h exposure: 4.3 ± 2.7%) and microfluidic cultures (5-h exposure: 5.6 ± 2.2%). These results further demonstrate the utility of our microfluidic device-culturing approach in capturing the different subpopulations of bacteria, differing in their growth and metabolic ability or phenotypic damage^12,34^.

### Ampicillin killing is independent of single-cell growth rate

**Fig. 4:**
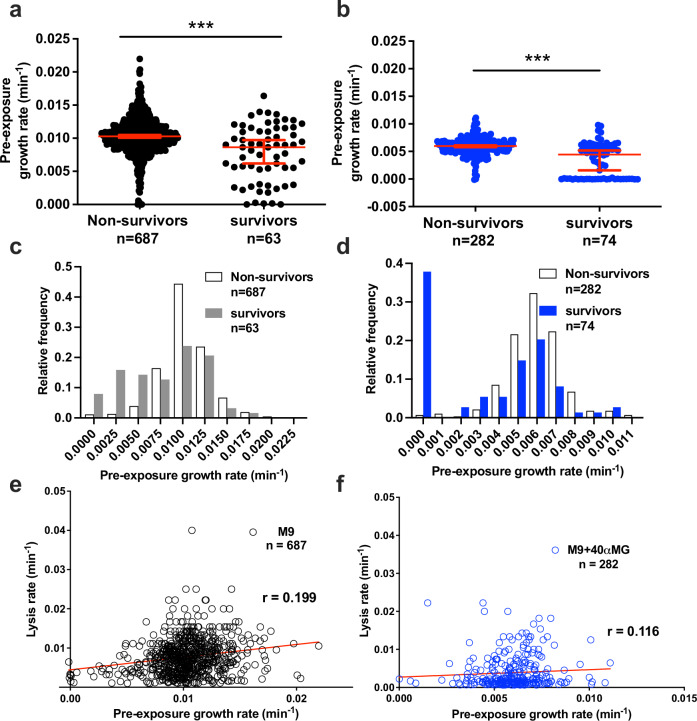
Growth inhibition is not essential for survival against ampicillin. Comparison of pre-exposure growth rates between non-survivors and survivors in M9 (**a**) or in M9+40X αMG (**b**). Each symbol depicts the pre-ampicillin growth rate of individual CFT073 cell and red bars indicate median ± 95% confidence intervals; asterisks (***) indicate significance (*p* < 0.0001, Mann–Whitney test). Relative frequency distribution of pre-exposure growth rates of non-survivors (white) and survivors (gray/blue), grown in M9 (**c**) or in M9+40X αMG (**d**). Correlation between pre-exposure growth rate and lysis rates amongst non-survivors cultured in M9 (**e**) or in M9+40X αMG (**f**). Each symbol represents a single-cell. The red-line represents the linear fit. The computed Spearman correlation coefficient value (*r*) is shown.

Significant heterogeneity was observed in both growth and lysis rates at the single-cell level. Consistent with population-level trends, the mean growth and lysis rates were reduced upon supplementation of M9 medium with αMG (Fig. S6). At the ensemble level, the pre-ampicillin exposure growth rates of survivors were slightly lower than the non-survivors when the CFT073 were cultured in M9 (median growth rate 0.009 vs 0.01 min^−1^) or in M9+40X αMG (median growth rate 0.004 vs 0.006 min^−1^) (Fig. 4a, b; Movie S6–S11). However, the distribution of elongation rates amongst the survivors overlapped with those of non-survivors (Kolmogorov–Smirnov test to compare distributions: M9: *P* = 0.76; M9+40X αMG *P* = 0.85) (Fig. 4c, d). To evaluate if slow-growing cells were more likely to survive killing by ampicillin, we also measured the lysis rates at the single-cell level and found very weak correlation between the pre-exposure growth rate and lysis rates under both the growth conditions (Spearman correlation coefficient *r* = 0.199 and 0.116 for bacteria grown in M9 and M9+40X αMG, respectively) (Fig. 4e, f). Interestingly, survivors originated from growing (Movies S6 and S7) as well as non-growing cells (Movies S8 and S9) independent of the growth condition (Fig. 4c, d). However, the majority (M9: 88.7%; M9+40X αMG: 73.3%) of cells that survived and regrew after 5 h of ampicillin treatment were growing and dividing normally prior to ampicillin exposure (Fig. S7). Lysis of non-growing cells was also observed (Movies S10 and S11). As in case of LB medium (Fig. 2b), most of the non-growing cells (71.4%) lysed on exposure to ampicillin in M9 medium, while 74.3% of non-growing cells survived when M9 was supplemented with αMG. Therefore, being in non-growing was not essential or protective against killing by ampicillin. The timing of awakening from the non-growing state did not affect the fate of the cells (Fig. S8).

### RpoS is not required for survival against ampicillin in exponentially growing cells

**Fig. 5:**
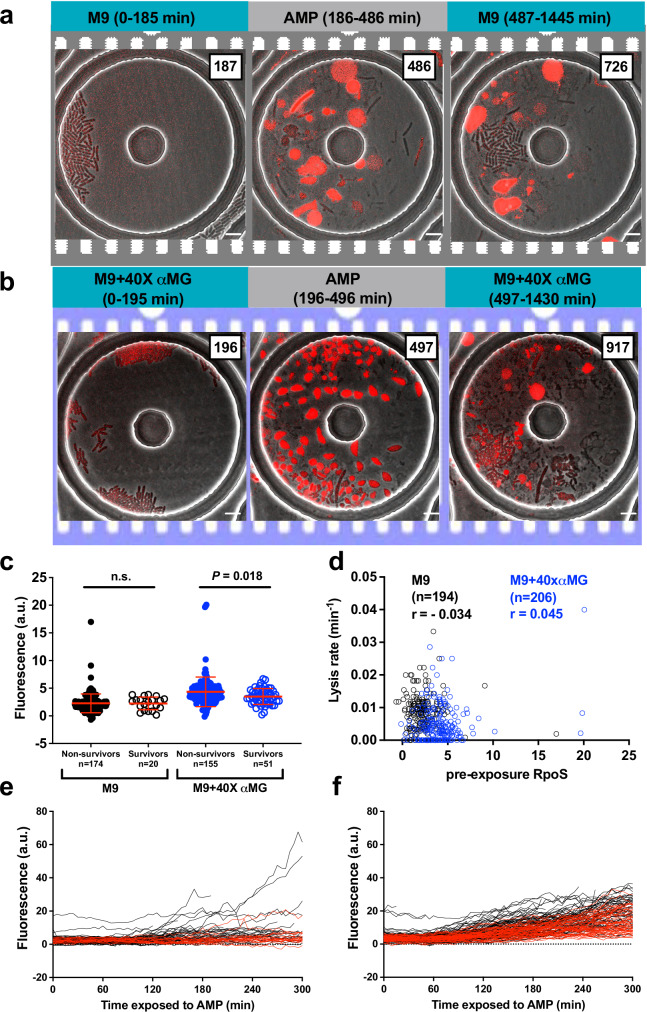
RpoS levels do not contribute to survival against ampicillin. Representative image series of ampicillin (AMP) -treated CFT073 carrying *rpoS-mCherry* in M9 (**a**) and M9+40X αMG (**b**). Exponentially growing cells were introduced into a microfluidic device and imaged on fluorescence and phase channels at 5 min intervals and exposed to 50 μg/ml ampicillin for 5 h. This experiment was repeated 2 times in each medium. Scale bar, 5 μm. **c** Comparison of pre-exposure RpoS-mCherry levels between non-survivors and survivors. Points depict single-cell RpoS-mCherry levels before ampicillin treatments and red bars represents means ± SD. *P* = 0.018 Mann–Whitney *t*-test. **d** Relationship between pre-exposure RpoS-mCherry levels and lysis rate in M9 (black circle) and in M9+40X αMG (blue circle) at single-cell level. The Spearman correlation coefficient value (*r*) is shown. Time traces of RpoS-mCherry levels during ampicillin exposure in arbitrary units for non- survivors (black) and survivors (red) in M9 (**e**) and in M9+40X αMG (**f**).

Exposure to stressful environments is associated with elevated levels of the alternate sigma factor, *σ*^S^ (RpoS) in bacteria^35–37^. RpoS levels are regulated by the signaling molecule (p)ppGpp, a key mediator of the starvation response that has also been implicated in promoting persistence through activation of the toxin-antitoxin systems in ampicillin-stressed *E. coli*^38,39^. To examine the role of RpoS in UPEC survival, we first measured RpoS expression levels. When growth rate was reduced by addition of αMG, exponentially growing cells expressed higher amounts of RpoS (Fig. S9a). We hypothesized that enhancement of RpoS levels might contribute to survival against antibiotic exposure. To test this hypothesis and to measure RpoS expression at the single-cell level, we constructed a strain in which the native copy of *rpoS* gene was replaced by *rpoS-mCherry* allele encoding for the fusion protein. We wanted to address if variation in (p)ppGpp and consequently RpoS-mCherry, prior to ampicillin exposure, had an influence on killing by the antibiotic. Cells expressing the fusion protein were harvested from exponential cultures and were cultured in a microfluidic device, and RpoS-mCherry expression levels monitored using time-lapse microscopy before and after ampicillin exposure (Fig. 5a, b, and Movies S12 and S13). Reduction of growth rate through αMG supplementation led to increased single-cell RpoS-mCherry expression which was accompanied by decreased lysis rates during ampicillin exposure (Fig. S9b, c). Unexpectedly, pre-ampicillin RpoS-mCherry levels were comparable between survivors and non-survivors, and were in fact lower in survivors in M9+40X αMG group (Fig. 5c). We did not observe any correlation between pre-exposure RpoS-mCherry levels and the lysis rates upon ampicillin treatment under both the growth conditions (Spearman correlation coefficient *r* = −0.034 and 0.045 for cells grown in M9 and M9+40X αMG, respectively) (Fig. 5d). We noted a slow increase in RpoS-mCherry levels upon exposure of cells to ampicillin (Fig. 5e, f). Since the timing of induction was variable across individual cells, we carried out analysis of RpoS-mCherry levels in survivors vs non-survivors at 1-h intervals after ampicillin exposure. We did not find any difference in RpoS-mCherry levels in these two populations when grown in M9 or M9+40αMG (Fig. S10a, b). However, it is important to note that recent studies have indicated that translational fusions of RpoS may be functionally defective^21,40^. Therefore, while the pre-exposure heterogeneity in RpoS-mCherry could be significant, the downstream activation might be compromised. To further validate our observations on single-cell RpoS levels not being an important factor for survival against ampicillin treatment in exponentially growing UPEC, we constructed a mutant strain of CFT073 (Δ*rpoS*) in which the *rpoS* allele was deleted. There was no significant difference in the ampicillin kill-kinetics of WT and Δ*rpoS* cells in either fast- or slow-growth conditions or during stationary phase (Fig. S11). We confirmed that bacteria in stationary phase or in a non-growing state, expressed higher RpoS levels compared to actively growing cells (Fig. S12). Thus, while single-cell RpoS levels correlate with the growth state, they do not correlate with persistence against ampicillin in CFT073.

### Efflux activity does not contribute to survival against ampicillin

**Fig. 6:**
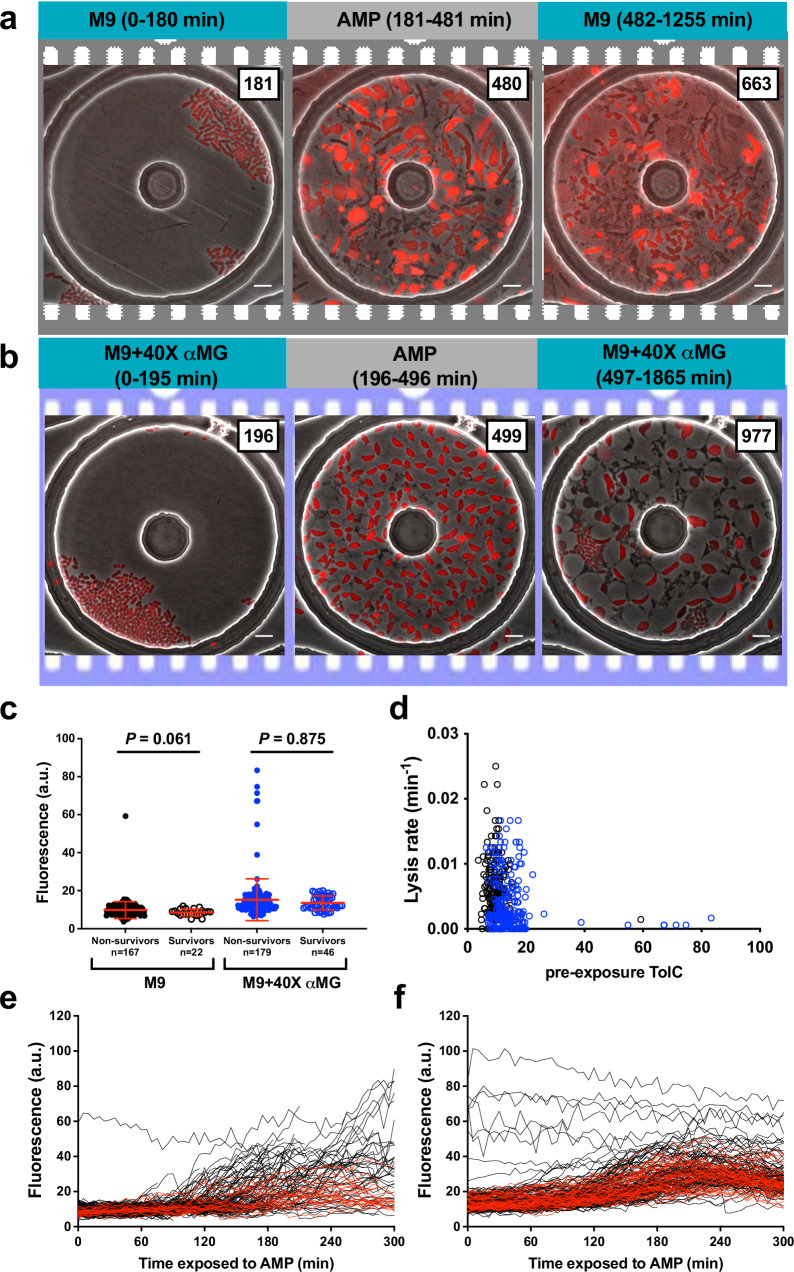
Single-cell TolC levels is not involved in cell fates. Representative image series of ampicillin (AMP) -treated CFT073 carrying p*tolC-mCherry* in M9 (**a**) and M9+40X αMG (**b**). Exponentially growing cells of p*tolC-mCherry* were introduced into a microfluidic device and imaged on fluorescence and phase channels at 5 min intervals and exposed to ampicillin at hours 3–8. This experiment was repeated 2 times in each medium. Scale bar, 5 μm. **c** Comparison of pre-exposure pTolC-mCherry levels between in M9 and M9+40X αMG, and between non- survivors and survivors. Points show single-cell pTolC-mCherry levels before ampicillin treatments and red bars indicate means ± SD. *P* = 0.061 and *P* = 0.875 Mann–Whitney *t*-test. **d** Relationship between pre-exposure pTolC-mCherry levels and death rate in M9 (black circle) and in M9+40X αMG (blue circle) at single-cell level. Time traces of pTolC-mCherry during ampicillin exposure in arbitrary units for non- survivors (black) and survivors (red) in M9 (**e**) and in M9+40X αMG (**f**).

An earlier study demonstrated that persister formation in nonpathogenic *E. coli* could be attributed to stochastic fluctuations in efflux activity^41^. The energy-dependent efflux pump AcrAB-TolC is known to transport various substrates, including antibiotics, reducing intracellular drug accumulation and facilitating bacterial survival under drug stress^42^. In fact, deletion of *tolC* or *acrAB* in *E. coli* results in strains with increased susceptibility to various antibiotics, including β-lactams^43,44^. We investigated the relationship between efflux activity and ampicillin killing at the single-cell level using an engineered strain in which wild-type chromosomal *tolC* allele was replaced by a p*tolC-mcherry* transcriptional fusion allele. Before initiating single-cell imaging of this strain, we wanted to confirm the functionality of the transcriptional fusion. Using the fluorescence probe Hoechst (H) 33342, a known substrate for AcrAB-TolC^45^, we were able to confirm that p*tolC-mcherry* levels correspond to efflux activity of CFT073 (Fig. S13a). Bacteria cultured in M9 had higher H33342 accumulation and low mCherry fluorescence, whereas supplementation of αMG resulted in an increase in *ptolC-mCherry* expression and correspondingly reduced accumulation of H33342 (Fig. S13a). These results support the hypothesis that high expression levels of *ptolC-mCherry* represent high efflux activity. Next, exponentially growing cells of this transcriptional reporter strain were cultured and imaged in our microfluidic setup, where they were exposed to ampicillin over a period of 5 h to assess whether *tolC* expression levels correlate with survival (Fig. 6a, b, and Movies S14 and S15). Single-cell analysis of cells expressing p*tolC-mcherry* fusion revealed that mCherry levels were enhanced upon supplementation of αMG (Fig. S13b), as seen before in the H33342 accumulation assay. We also confirmed that the constructed reporter strain exhibited lower lysis rates upon ampicillin exposure when the growth rate was reduced by αMG (Fig. S13c). However, the pre-ampicillin *ptolC-mCherry* levels in survivors and non-survivors were found to be comparable (Fig. 6c), with no significant correlation between *ptolC-mCherry* levels and lysis rates (Fig. 6d). Exposure to ampicillin, resulted in heterogenous induction of *ptolC-mCherry* in a subset of cells under both growth conditions (Fig. 6e, f), however, single-cell analysis of post-ampicillin *ptolC-mCherry* levels did not reveal any differences between survivors and non-survivors (Fig. S14). These results confirm that while efflux activity might contribute to decreasing susceptibility to antibiotics at the population level, it does not appear to be a significant factor influencing survival of individual cells against ampicillin.

## Discussion

Bacterial persistence, a phenomenon distinct from antibiotic resistance, refers to the ability of a small subpopulation of genetically identical bacterial cells to survive normally lethal concentrations of antibiotics. These persisters are phenotypic variants that upon removal of the antibiotic can resume growth and generate new populations that remain susceptible to the antibiotic. Persistence is widely recognized as a major barrier to clearing bacterial infections and a significant contributor to antibiotic treatment failures, recurrent infections, and the evolution of classical genetic resistance. A prominent example are UTIs, which are predominantly caused by UPEC and are prone to recurrence, highlighting the need for improved antimicrobial therapies.

For decades, the prevailing hypothesis has attributed persistence to a dormant or non-growing physiological state, often termed “triggered persistence”, where cells enter this state prior to antibiotic exposure due to stressful environmental conditions like nutrient deprivation or stationary phase^46,47^. It was postulated that this dormancy protects bacteria because most antibiotics target active cellular processes and are less active on non-growing cells^14^. Recent advancements, particularly in single-cell analysis techniques, are increasingly challenging the singular “dormancy” paradigm, revealing a more complex and heterogenous landscape of persister states. Recent study generated high-resolution single-cell RNA atlases of *E. coli* growth transitions and found that persisters from diverse genetic and physiological models converge to transcriptional states distinct from standard growth phases, exhibiting a dominant signature of translational deficiency^48^. In *Bacillus subtilis*, a recent study highlighted the role of the alarmone, (p)ppGpp, in promoting persistence through the depletion of intracellular GTP and affecting processes like protein translation and DNA synthesis^49^. These findings are in line with previous research suggesting that persisters are low-energy cells, characterized by lower ATP levels^50,51^. However, contrary to the dormancy hypothesis, accumulating evidence indicates that actively growing cell fractions can also generate persister cells^16–21^. Single-cell observations of *E. coli* exposed to ampicillin or ciprofloxacin showed that most persisters were actively growing before antibiotic treatment^21^. These “growing persisters” exhibited heterogeneous survival dynamics, including continuous growth and fission with L-form like morphologies, responsive growth arrest, or post-exposure filamentation, suggesting that multiple survival modes can coexist within a single persister population. In this work, we investigated if the natural variation in growth rates observed in exponentially growing bacteria has an influence on ampicillin persistence in a clinical isolate of UPEC. Our data clearly demonstrated that the bacteria that survived antibiotic exposure were growing and dividing at similar rates as the non-survivors. While a small minority of persisters (16.6%) were indeed in a non-growing state, being in a non-growing or slow-growing state was not correlated with survival. To further delineate the role of growth rate, UPEC was cultured under nutrient-rich and nutrient-limiting conditions using αMG, a non-metabolizable glucose analog that competitively inhibits glucose uptake, thereby reducing growth and cell division without triggering starvation responses. While at the population level slow growth mediated by αMG supplementation was associated with higher survival frequencies, our single-cell analyzes demonstrated very weak correlation between growth rates and survival. Under both nutrient-rich and nutrient-limited (M9+40αMG) conditions the growth rate distribution of survivors completely overlapped with that of non-survivors.

Besides growth rate, other factors have been implicated in persister formation, for example, Toxin-Antitoxin systems, SOS response, efflux pumps and L-forms. Hong et al. showed that deletion of *rpoS* dramatically promotes *E. coli* persister formation in the presence of ampicillin by rendering cells dormant via overexpression of the toxin-antitoxin system^52^. In our study, while conditions that elevated RpoS expression were associated with higher overall survival probabilities at the population level (Fig. S9), no correlation was observed between single-cell RpoS-mCherry and survival outcomes. Moreover, deletion of *rpoS* had no measurable effect on persistence in UPEC cultured in M9-based media (Figs. 5 and S11). These results differ from those observed in MG1655, where the loss of *rpoS* was linked to a reduced fraction of persisters or a change in the decay rate^21^.

An active defense strategy employed by growing bacteria to survive antibiotic exposure is through activation or overexpression of drug efflux pumps like AcrAB-TolC, which reduce intracellular antibiotic concentrations^41,53^. We observed that the average survival probability of the population is higher under conditions where efflux pump activity (and correspondingly p*tolC-mcherry*) levels were elevated. However, there was no correlation between p*tolC-mcherry* expression and survival probability at the single cell level (Fig. 6). This finding contrasts with previous reports on the persistence of wild-type *E. coli* (BW25113) to carbenicillin, where persister cells exhibited higher mean efflux activity and elevated TolC expression levels compared to sensitive cells^41^. Differences in the experimental conditions such as antibiotics, strains, could be responsible for the differences observed in the survival modes. Additionally, it is important to note that in the previous study, survivors were also observed within the subpopulation exhibiting low efflux activity at single-cell level, indicating that elevated efflux alone was not a strict requirement for persistence^41^. Bergmiller et al. reported that, under low concentrations of antibiotics, mother cells inheriting the old pole exhibited higher efflux activity than daughter cells inheriting the new pole^54^. This was attributed to the asymmetric distribution of drug efflux pumps, during cell division, favoring sustained growth of mother cells at subinhibitory antibiotic concentrations. In contrast, in our studies using high antibiotic concentrations, we did not observe any association between pole age and survival.

A distinct and increasingly recognized form of persisters includes cell-wall-deficient bacteria, such as L-forms. These have been observed in vitro and in vivo, often in response to β-lactam antibiotics, and can resume growth upon antibiotic removal. Interestingly, in this study, some of the survivors during and after ampicillin exposure exhibited radical morphological changes and transitioned to L-form or amoeboid shapes. Some of these giant amoeboid forms were observed to undergo fragmentation, with certain fragments capable of regrowing and dividing to produce normal rod-shaped cells. While we cannot completely dismiss the possibility that the microdevice’s structure plays a role, as shown by a previous study on *E. coli* passing through submicron constrictions^55^, it is likely that the observed shape changes contribute to *E. coli* survival in the presence of ampicillin. Similar shape alterations have been reported with ampicillin and clinical isolates and lab strains of *E. coli*^21,29,31,56^, and L-form cells have been isolated as β-lactam tolerant cells from infected humans and animals^57^. It has been suggested that formation of cell-wall deficient forms while on the one hand aids the bacteria to survive cell wall targeting stresses such as β-lactams, on the other hand makes it more susceptible to osmotic and other stresses. These L-forms are likely responsible for the discrepancies often observed between survival fractions determined by CFU-based plating methods and real-time microscopy. CFU assays involve washing steps and recovery on solid media following antibiotic exposure, which can induce drastic nutritional shifts and additional stress, potentially leading to cell death not directly caused by the antibiotic itself^11,56^. In contrast, the MPN assay is less harsh, as it avoids extensive washing and does not require growth on solid media. Also, it can be performed using the same media conditions as those used during the antibiotic exposure. Consistent with this, we observed greater concordance in survival estimates between our real-time single-cell microscopy approach and the MPN assay, as recently reported in Salmonella as well^11^.

In UTIs, clusters of UPEC bacteria persist as quiescent intracellular reservoirs (QIRs) within the urinary bladder epithelium. These QIRs are considered to be the major cause of recurrent UTIs^58–60^. Formation of QIRs is preceded by active growth and replication of bacterial population forming intracellular bacterial communities followed by penetration into deeper layers to form QIRs^59,61^. Their intracellular niche along with their quiescent physiological state allows the bacteria to resist treatment with antibiotics and evade host immune responses and resembles the antibiotic-tolerant persister state observed in dormant bacterial populations. While both quiescent reservoirs and persister subpopulations can contribute to recurrent UTIs and treatment failures, these two states have also been shown to be metabolically distinct^60^.

Our observations support the emerging consensus that persistence is not exclusively linked to a dormant state but can arise from plural dynamic responses of bacterial cells to antibiotic stress, which align with recent studies^11,16,17,19,21^. Accurately studying persister cells is inherently difficult due to their low frequency and transient nature. The presence of multiphasic kill curves suggests that different mechanisms may operate at different time scales of survival (hours, days, weeks). To overcome these challenges, researchers are increasingly relying on real-time, single-cell assays, such as microfluidic devices, which provide direct observation of individual cell histories before, during, and after antibiotic treatment. Furthermore, novel tools like pSCRATCH have been developed to heritably mark persisters during their transient growth arrest, allowing researchers to track the progeny of persisters and directly investigate their role in infection relapse^62^. The profound clinical implications of bacterial persistence necessitate a deeper understanding of these diverse survival strategies. While some studies suggest limited interference from persisters in short treatments for non-resistant bacteria, longer treatments for difficult infections like tuberculosis, UTI or *Staphylococcus aureus* infections highlight their relevance. The ability of persisters or tolerant subpopulations to facilitate the evolution of resistance underscores the urgent need to address this challenge. Future research must focus on integrating single-cell techniques with microtissue models to characterize the full spectrum of persister mechanism. This comprehensive understanding is essential for developing novel anti-persister compounds, such as those targeting energy-independent cellular processes, or implementing antibiotic dosing strategies to prevent infection relapse and combat the rising threat of antimicrobial resistance.

## Methods

### Bacteria, strain construction and growth conditions

Uropathogenic *E. coli* (UPEC) strain CFT073^24^ was provided by H. Mobley, University of Michigan, USA. Enterohemorrhagic *E. coli* (EHEC) strain CIP 105917, serotype O157:H7 was provided by the Centre de Resources Biologiques, Institut Pasteur, France. The lab-adapted *E. coli* strain MG1655 was provided by S. Leibler (The Rockefeller University, New York). To facilitate microscopy and image analysis, all strains were transformed with a plasmid (pZA32-YFP) expressing YFP from a strong promoter^63^. Cells were grown in Luria-Bertani (LB) Miller medium (Sigma) or in M9 minimal medium containing 2 g l^−1^ glucose, 1 M MgSO_4_, and 1 M CaCl_2_ (M9) at 37 °C with shaking (180 RPM) until exponential phase, and aliquots were stored in 15% glycerol at −80 °C. Aliquots were thawed, used once, and discarded.

Reporter strains were constructed using a modified version of the method described previously^64^. Cells were transformed with pKD46, which carries the arabinose-inducible λred gene. CFT073 cells containing pKD46 were cultured in LB medium at 30 °C. To induce the λred gene, overnight cultures were diluted 20-fold in 10 ml of fresh LB supplemented with 0.35% arabinose, and incubated for 2 h until reaching an A_600nm_ ~ 0.8. For a chromosomal *rpoS-mcherry* translational fusion, cells were electroporated with a purified PCR product amplified from pUC19 encoding kanamycin and mCherry genes, using rpoS-mch-F (GCAAACGCAGGGGCTGAATATCGAAGCGCTGTTCCGTGAG AGTGATTTT ATGGTGAGCAAGGGCGAGGAGGATA) and rpoS-mch-R (CAGCCTCGCTTGAGACTGGCCTTTCTG ACAGATGCTTACTCCTCCTTAGTTCCTATTCC) as primers and plated on LB agar containing kanamycin (50 μg ml^−1^). The primers were designed to generate a small Ser-Asp-Phe-Met peptide linker between the C-terminal part of RpoS and the N-terminal part of mCherry. After the transduction of rpoS-mcherry-frt-aphA-frt recombinant allele, aphA resistance cassette was removed using pCP20^64^. A chromosomal p*tolC-mcherry* transcriptional fusion was constructed as described above using PCR product amplified from pUC19 using tolC-mch-F (GTTTTATCCGCATAT TTTTGTTGAGTAAAAGGAGGATAAACATATGGTGAGCAAGGGCGAGGAGGATA) and tolC-mch-R (GCTTATCGGGGCAATATTAAGCTGTATCCTCCTTAGTTC CTATTCC) as primers. After transduction of mcherry-frt-aphA-frt recombinant allele, aphA cassette was removed, using pCP20^64^. To construct rpoS deletion mutant in CFT073, the FRT-flanked kanamycin resistance cassette in pKD4^64^, was amplified using the primers CFTrpoSUp (CGTCAAGGGATCACGGGTAGGAGCCACCTTATGAGTCAGTGTGTAGGCTGGAGCTGCTTCG) and CFTrpoSDn (CGCTTGAGACTGGCCTTTCTGACAGATGCTTACTTACTCCATATGAATATCCTCCTTAG). The purified PCR product was digested with *Dpn*I and was electroporated into induced CFT073 cells pKD46 and plated on LB-agar containing 50 μg ml^−1^ kanamycin. Deletion was confirmed by PCR. The kanamycin resistance cassette was eliminated from the mutant strain using the helper plasmid pCP20^64^. All strains were verified by sequencing and functional assays.

### Time-kill assays in LB medium

Frozen bacterial stocks were thawed, diluted 1:100 in LB medium, and incubated overnight at 37 °C / 180 RPM. Overnight cultures were diluted 1:1000 in prewarmed LB medium and incubation was continued until an optical density at 600 nm (A_600 nm_) of 0.01 was reached. Aliquots were serially diluted and plated to determine the starting bacterial density (0 min) just before addition of 50 μg ml^−1^ ampicillin (Sigma) and incubation was continued. At 30, 60, 90, 120, 150, 180, 240, and 360 min after ampicillin addition, aliquots were withdrawn, washed, serially diluted in prewarmed LB medium, and plated on LB agar (Sigma). CFU were counted after incubating the plates overnight at 37 °C.

### Time-kill assays in M9 medium

Frozen stocks of bacteria cultured in M9 were thawed and diluted 1:5000 in M9 medium. Cultures were grown at 37 °C to mid-exponential phase (A_600 nm_ ~ 0.2) and used directly for the killing assay. For experiments under slow-growth conditions, mid-exponential cultures grown in M9 were diluted 1:500 and re-inoculated in M9 supplemented with 80 g l^-1^ αMG (M9+40X αMG) and grown to mid-exponent−ial phase (A_600 nm_ ~ 0.1). Exponentially growing cells were exposed to 50 μg ml^−1^ ampicillin (Sigma). Aliquots were withdrawn at pre-determined time points, collected by centrifugation, washed with the corresponding medium, serially diluted and plated on LB agar. CFU were counted after incubating the plates overnight at 37 °C. MPN assays were performed in triplicates in 48 well plates, by serially diluting 50 μl of washed cell suspensions in 450 μl of corresponding medium. MPN counts were calculated with 95% confidence limit as per the FDA procedures^33^.

### MIC assays

Frozen bacterial stocks were thawed, diluted 1:100 in LB medium, and incubated overnight at 37 °C/180 RPM. MICs were determined using the broth microdilution technique. Briefly, overnight cultures were diluted to A_600 nm_ 0.005 in 2 ml of prewarmed LB medium containing twofold serial dilutions of ampicillin in 14-ml round-bottom tubes (BD biosciences), then incubated at 37 °C / 180 RPM for 18 h. The MIC was defined as the lowest ampicillin concentration that inhibited bacterial growth by at least 90% compared to the no-ampicillin control.

### Device fabrication

The microfluidic device assembly consists of a PDMS chip, a cellulose semi-permeable membrane, and patterned coverslips. Each component was prepared with slight modification of a previously described method^26,65^. The PDMS chip was made using standard soft lithography techniques. A silicon wafer was spin-coated with negative photoresist SU8 GM1075 (Gersteltec) to a thickness of 300 μm and exposed to UV light using a mask with a serpentine design. After development, the wafer was used as a mold to cast PDMS (1:10, curing agent:elastomer). The PDMS was cured at 80 °C and holes were punched in the 5 mm thick PDMS chip using a 2 mm-diameter puncher. Silicon tubes (0.76 mm, inner diameter) were connected at the inlet and outlet and sealed using PDMS as glue. Cellulose semi-permeable membrane with molecular weight cut-off of 25 kDa, was washed extensively with bi-distilled water and stored desiccated. Circular sections 25 mm in diameter, were rehydrated with the respective culturing medium before use by placing them on wet filter paper. The patterned coverslip, membrane, and PDMS chip were sandwiched in a custom made PMMA holder^65^.

### Fabrication of patterned coverslips

Patterned coverslips were fabricated using the clean room facilities at EPFL’s Center of MicroNanoTechnology. Borosilicate 25 mm plasma-treated #1 coverslips (Menzel-Glazer) were spin-coated with SU8 GM1040 (Gersteltech), baked, and exposed to UV light at 150 mJ/cm^2^ through custom-made masks^26^. After removing non-exposed photoresist with propylene glycol monomethyl ether acetate, the coverslips were extensively washed and dried. The resulting structures on the coverslips ranged from 0.8 to 1 μm in height.

### Time-lapse microscopy

Single-cell imaging was carried out as described^34,65^. In brief, a frozen stock of bacteria was thawed, diluted 1:100 in LB medium and incubated overnight at 37 °C/180 RPM. The overnight culture was diluted to A_600 nm_ 0.005 in prewarmed LB medium and incubation was continued until the culture reached A_600 nm_ 0.1. In case of M9, cells from frozen stocks were diluted 1:5000 and grown to mid-log phase (A_600 nm_ 0.1–0.2). For the experiments under slow-growth conditions, 50 μl of mid-exponential cultures grown in M9 were re-inoculated into M9+40X αMG and allowed to grow to mid-exponential phase (A_600 nm_ 0.1). A 3–5 μl aliquot of culture was inoculated between a patterned coverslip and a semipermeable membrane, which was overlaid with a polydimethylsiloxane chip patterned with microchannels for medium flow. This assembly was clamped in an acrylic frame and mounted on the stage of an inverted fluorescence microscope (Applied Precision PersonalDV, GE Healthcare) equipped with an environmental chamber at 37 °C. Images were acquired using a 100X oil-immersion objective (Olympus Plan Semi Apochromat; NA 1.3) and a CoolSnap HQ2 camera running Softworx software (GE Healthcare). Medium ± 50 μg ml^−1^ ampicillin was pumped through the microfluidic device at a flow rate of 25 μl min^−1^. In a typical experiment, 25–35 chambers, initially containing 1–5 cells, were selected for imaging. Images were acquired every 4 min on the phase-contrast and FITC (Ex 490 nm/Em 528 nm) or YFP (Ex 500 nm/Em 535 nm) fluorescence channels over a period of 24 h. In case of M9 media, images were acquired at 5–10 min interval.

### Image analysis

Images were analyzed using ImageJ v1.47n or Oufti^66,67^. For measurements of single-cell growth rates, projected cell area was measured using the polygon function and plotted over time. The increase in cell size was fitted to an exponential curve to derive the growth rate. The change in cell number over time (Fig. 1) was determined using the Cell Counter plug-in of ImageJ. To calculate killing rates, the number of lysis events between successive time points (4-min intervals) was counted and normalized by the total cell number at that time point. These values were divided by the time interval (4 min) to determine killing rates.

### Western blotting

Cells were harvested from either exponential or stationary phase and collected by centrifugation. Cell pellets were resuspended in lysis buffer (10 mM Tris-Cl pH 7.5, 150 mM NaCl, 0.5 mM EDTA, 0.5% glycerol), supplemented with 1X sample buffer (NuPAGE LDS, Invitrogen) and boiled at 100 °C for 10 min. Proteins were separated on NuPAGE 4–12% Bis-Tris gels (Invitrogen), and electro-transferred to nitrocellulose membrane. Membranes were incubated overnight at 4 °C with TBS 1X buffer (BioRad) containing 0.05% Tween-20 (Fisher Scientific) (TBS-T) and 5% non-fat milk (Applichem). Membranes were then incubated for 1 h with purified mouse anti-*E. coli* sigma S (anti-RpoS antibody) (clone 1RS1, BioLegend) or with purified mouse anti-*E. coli* sigma A (anti-RpoD antibody) (clone 2G10, BioLegend), both at 1:8000 in TBS-T+1% milk at 4 °C. Membranes were washed three times for 5 min in the same buffer at 4 °C, before being incubated for 45 min with a rabbit anti-mouse immunoglobulin conjugated to horseradish peroxidase (Dako, 1:3000) in TBS-T+1% milk at 4 °C. After three washes with TBS-T, membranes were incubated with HRP chemiluminescent substrate (mix of Super Signal West Pico from ThermoFisher and CPS-1 from Sigma-Aldrich, 2:1), wrapped in Saran film and exposed in a dark room in an autoradiography cassette against ECL film (GE Healthcare).

### Efflux activity assay

Efflux activity was measured as described previously^45^, using Hoechst (H) 33342, which accumulates in AcrAB-TolC deficient cells. Cells carrying a p*tolC-mcherry* transcriptional fusion were grown to mid-log phase and collected by centrifugation and resuspended in PBS to ~10^9^ CFU/ml. Aliquots (180 μl) were transferred to wells of a 96-well flat-bottomed black plate (Greiner Bio-one). Fluorescence was read from the top of the wells using excitation and emission wavelengths of 587 and 610 nm, respectively, to quantify pTolC-mcherry protein levels. Then, H33342 (20 mM) was added (20 μl) to each well to give a final concentration of 2.5 μM. The fluorescence intensity of H33342 was read at excitation 355 nm and emission at 460 nm, at 1 min intervals for a total duration of 1 h. Fluorescence levels of *mcherry* and Hoechst 33342 were compared.

### Statistical analysis

Statistical analysis and data fitting were done using Prism software v7 (GraphPad). For estimating decay rates (*k*_*fast*_ and *k*_*slow*_) of cell populations, the cell-count curves were fitted to a two-phase exponential decay equation with the constraint that *k*_*fast*_ be at least 1.3 times greater than *k*_*slow*_.

## Supplementary information

Miyahara_Supplementary_data_version2

MovieS1

MovieS2

MovieS3

MovieS4

MovieS5

MovieS6

MovieS7

MovieS8

MovieS9

MovieS10

MovieS11

MovieS12

MovieS13

MovieS14

MovieS15

MovieS16

## Data Availability

Supplementary information is available for this paper. Correspondence and requests for materials should be addressed to N.D. or J.D.M. All the raw data and single-cell analyzes source data has been deposited in the figshare repository and can be accessed at 10.6084/m9.figshare.30780101.
