# Supplementary material for: Survival of ampicillin-treated uropathogenic Escherichia coli is independent of single-cell growth rates
Source: NPJ Antimicrob Resist. 2026 Feb 2;4:7. doi: 10.1038/s44259-025-00180-6 (PMC12864937; doi:10.1038/s44259-025-00180-6)
Supplement: Supplementary file 1 — Miyahara_Supplementary_data_version2 [file 44259_2025_180_MOESM1_ESM.docx]

Supplementary Data

Survival of ampicillin-treated uropathogenic *Escherichia coli* is independent of single-cell growth rates

Yoshiko Miyahara^1,2^**^#^**, François Signorino-Gelo^1¶^, Nicolas Elian Michel Lonchampt^1^, Paul Murima^1^, John D. McKinney^1^*, Neeraj Dhar^1,3,4,5^**^#^***

^1^Global Health Institute, School of Life Sciences, Swiss Federal Institute of Technology in Lausanne (EPFL), 1015 Lausanne, Switzerland

^2^R&D, Safety Science Research, Kao Corporation, Akabane, Ichikai, Haga, Tochigi, Japan

^3^Vaccine and Infectious Disease Organization (VIDO), University of Saskatchewan, Saskatoon, SK S7N 5E3, Canada

^4^Department of Biochemistry, Microbiology and Immunology, College of Medicine, University of Saskatchewan, Saskatoon, SK S7N 5E5, Canada

^5^Vaccinology & Immunotherapeutics Program, School of Public Health, University of Saskatchewan, Saskatoon, SK S7N 5E5, Canada

#- Equal contribution

¶ - Deceased

*Correspondence to: [john.mckinney@epfl.ch](mailto:john.mckinney@epfl.ch) or [neeraj.dhar@usask.ca](mailto:neeraj.dhar@usask.ca)


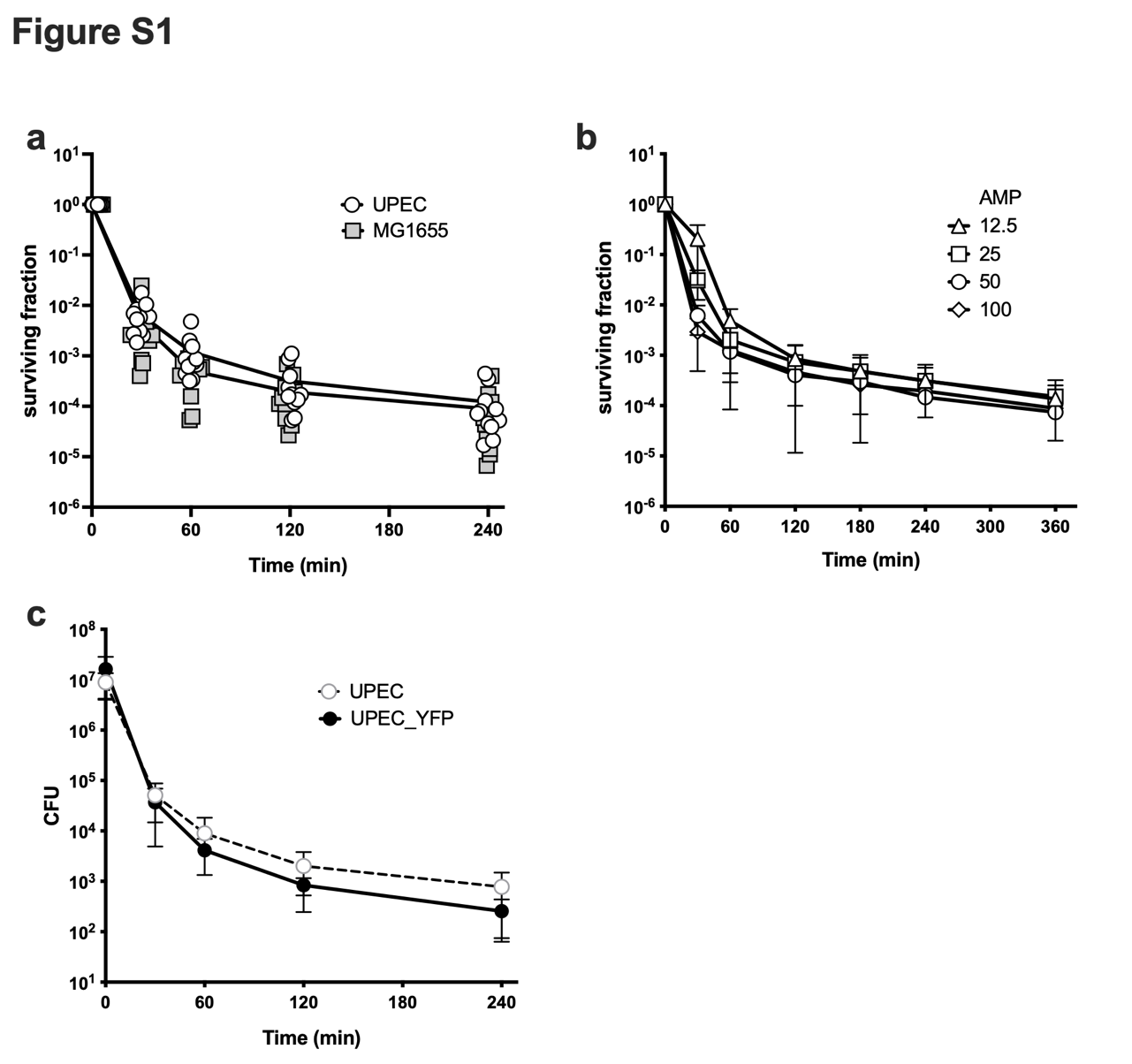


**Supplementary Figure S1. UPEC persistence during ampicillin exposure**.

**(a)** Time-kill kinetics of UPEC strain CFT073 (circles) and lab-adapted strain MG1655 (squares) exposed to 50 μg ml^-1^ ampicillin. At the indicated time points, aliquots of cells were plated on LB agar to measure colony-forming units (CFU). Solid line represents the mean of the data from twelve independent experiments. **(b)** Exponential-phase cultures of UPEC strain CFT073 were exposed to 12.5, 25, 50, or 100 μg ml^-1^ ampicillin, equivalent to ~ 2X, 4X, 8X, or 16X the minimum inhibitory concentration (MIC), respectively. At the indicated time points, aliquots of ampicillin-treated cells were washed and plated on LB agar to measure colony-forming units (CFU). Data are means + SD of five independent experiments. **(c)** Expression of yellow fluorescent protein (YFP) in UPEC does not affect the kinetics of killing by ampicillin. Exponential-phase cultures of UPEC strain CFT073 transformed with a YFP-expressing plasmid (solid circles) were exposed to 50 μg ml^-1^ ampicillin. At the indicated time points, aliquots of cells were washed and plated on LB agar to measure colony-forming units (CFU). Data are means + SD of three independent experiments. The data for UPEC strain CFT073 (white circles) has been replotted from Figure S1a for comparison.

**Supplementary Figure S2. Majority of the UPEC lineages that survive during ampicillin treatment are growing and dividing normally at the time of ampicillin exposure.** Line-plots depicting cell growth and division in lineages of UPEC strain CFT073 that survive during ampicillin treatment. Cells were cultured in a microfluidic device and individual cells were tracked by time-lapse microscopy before, during (grey shading), and after a 4-hour exposure to 50 μg ml^-1^ ampicillin. Magenta lines, old-pole cells. Green lines, new-pole cells. Black lines, generations before and after ampicillin. Blue asterisk, cell death. Data are from seven independent experiments.

#

**Supplementary Figure S3. Line-plot depicting cell growth and division in a surviving lineage of UPEC strain CFT073.** Cells were cultured in a microfluidic device and individual cells were tracked by time-lapse microscopy before, during (grey shading), and after a 6-hour exposure to 50 μg ml^-1^ ampicillin. Magenta lines, old-pole cells. Green lines, new-pole cells. Black lines, generations before and after ampicillin exposure. Blue asterisk, cell death.


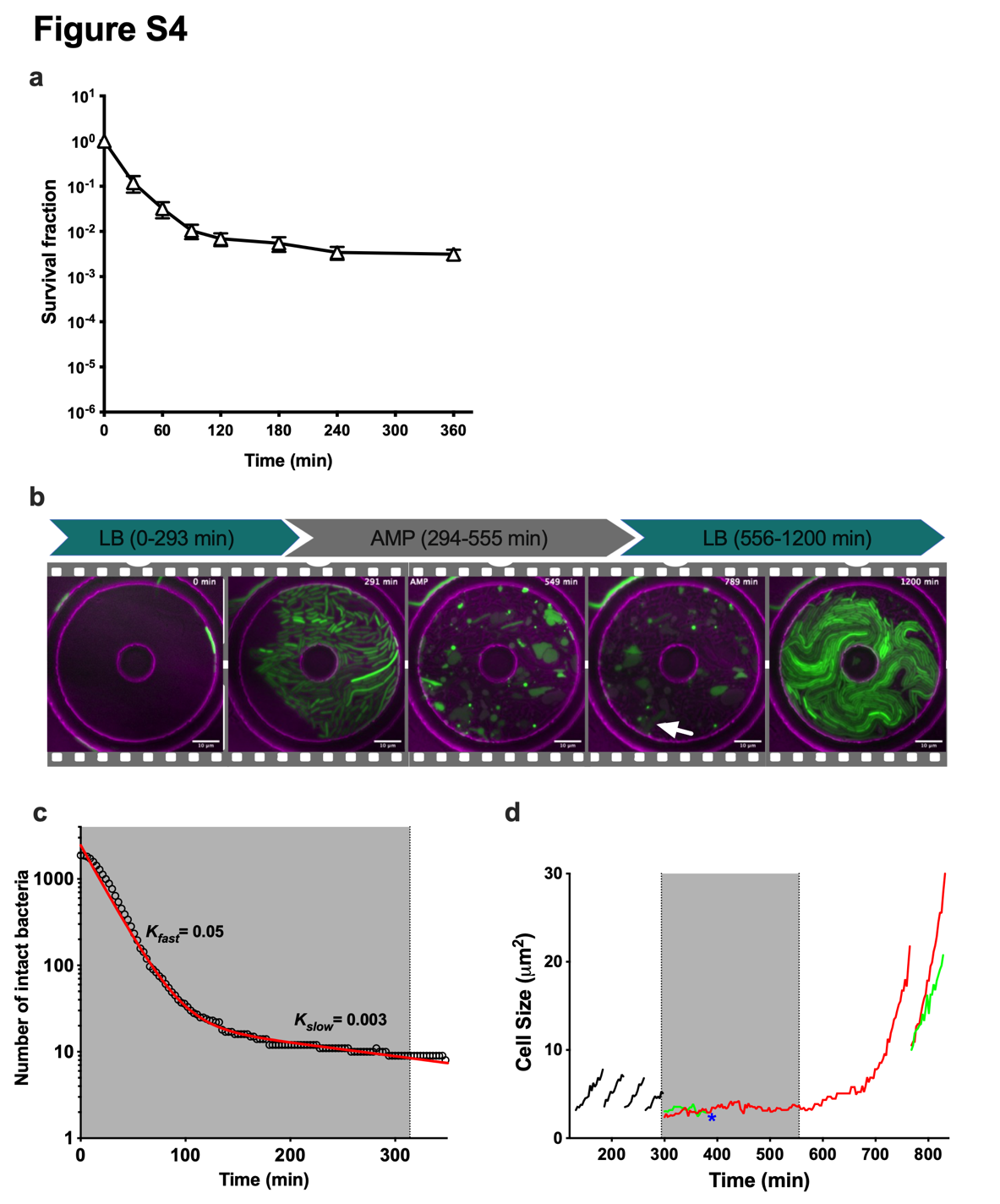


**Supplementary Figure 4. EHEC persistence during ampicillin exposure.**

**(a)** Time-kill kinetics of EHEC strain CIP 105917 (triangles) exposed to 50 μg ml^-1^ ampicillin. At the indicated time points, aliquots of cells were plated on LB agar to measure colony-forming units (CFU). Solid line represents the mean of the data from six independent experiments. **(b)** A representative series of time-lapse images of ampicillin-treated EHEC strain 105917 expressing YFP. The fluorescence (green) and phase (magenta) images are merged. Scale bar, 10 μm. Time (minutes) is indicated on the top right. Ampicillin (50 μg ml-1) was added to the flow medium at 294-555 minutes. In this example, one cell survives antibiotic exposure (white arrow) and repopulates the chamber after ampicillin withdrawal. **(c)** Surviving EHEC cells were enumerated by time-lapse microscopy during exposure to 50 μg ml-1 ampicillin. Data from one representative experiment. Five independent replicate experiments were carried out. Four persister cells were detected, of which 3 were growing prior to ampicillin exposure and one was a non-growing persister. Lysis kinetics over time of ampicillin-treated EHEC. The red line depicts the two-phase fitting of cell numbers over time to determine the *k_fast_* and *k_slow_* lysis rates. **(d)** Line-plot depicting cell growth and division in a lineage of an EHEC cell that survives during ampicillin treatment. Cells were cultured in a microfluidic device and individual cells were tracked by time-lapse microscopy before, during (grey shading), and after a ~4.5-hour exposure to 50 μg ml^-1^ ampicillin. Red lines, old-pole cells. Green lines, new-pole cells. Black lines, generations before ampicillin. Blue asterisk, cell death. This lineage is representative of the three persister cells that were growing prior to ampicillin exposure.


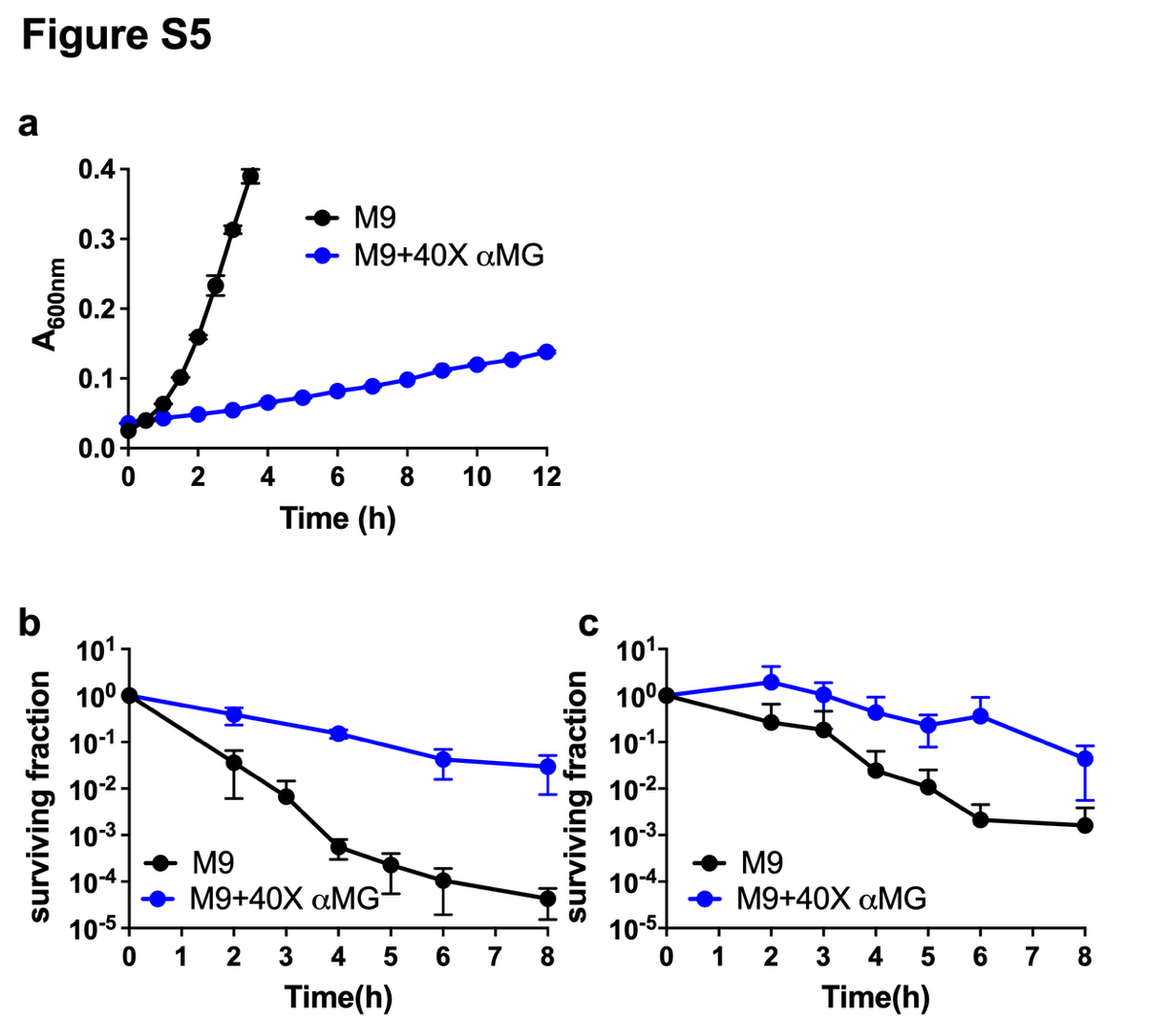


**Supplementary Figure S5. Supplementation of αMG affects the growth rate of UPEC. a,** Growth curves of CFT073 in M9 (M9; black) and in M9 supplemented with αMG (M9+40X αMG; blue). OD_600nm_, optical density (absorbance) at 600 nm. **b, c**, Killing kinetics against ampicillin in M9 (black) and in M9+40X αMG (blue) measured by the colony counting method **(b)** and by the MPN method **(c).** Exponentially growing cells in each medium were exposed to ampicillin (50 μg ml^-1^). In **(b)**, aliquots were removed at the indicated time points, washed, and plated to count survivors. In **(c)**, aliquots were removed at the indicated time points, washed, serially diluted with media to a degree such that inocula in the tubes will sometimes but not always contain viable cells, and cultured. Original concentrations of surviving cells were estimated from the number of tubes with growth at each dilution. Points represent means ± SD (*n* ≥ 3).


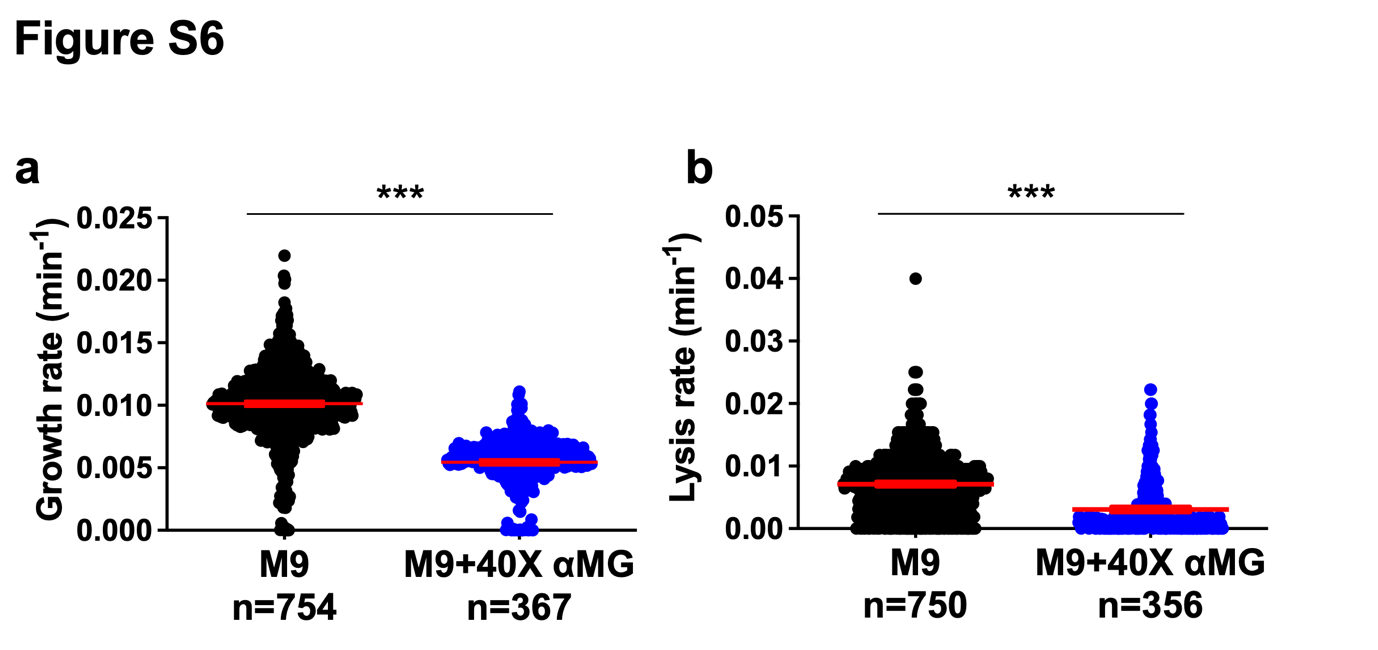


**Supplementary Figure 6. Distribution of pre-exposure elongation rates and lysis rates in slow-growth and fast-growth populations. a,b,** Comparison of single-cell elongation rates (**a**) and lysis rates against to ampicillin (50 μg ml^-1^) between M9 and M9 supplemented with 40X αMG. Dots represents single-cell elongation rates or lysis rates, and red lines indicate median ± 95% confidence intervals; asterisks (***) indicate significance (P<0.0001, Mann-Whitney test).

**Figure S7**

**M9 M9+40X** α**MG**

**Supplementary Figure 7. Non-growing survivors are rare. a**,**b**, Chart showing relative proportion of growing non-survivors (green), growing survivors (dark green), non-growing non-survivors (red), and non-growing survivors (dark red) in M9 (**a**) and in M9 supplemented with 40X αMG (**b**). Numbers indicate the percentage of each type of cells in all populations.


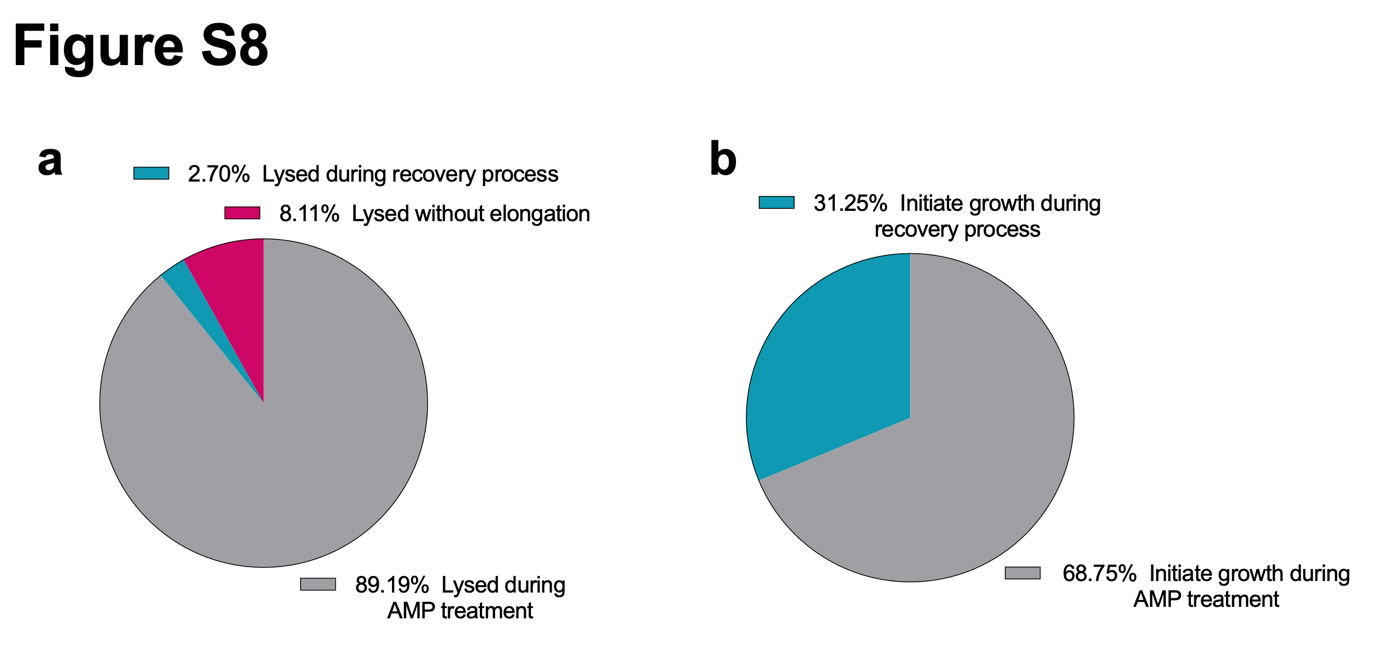


**Supplementary Figure 8. The timing of awakening from the non-growing state does not affect cell fates. a)** Chart shows the fate and the timing of regrowth for non-growing cells that eventually lyse upon treatment with ampicillin in M9. 8.1% of these cells lyse without elongation; ~89% initiate growth during ampicillin treatment and then lyse; ~2.7% initiate growth during ampicillin washout recovery phase and then lyse. **b)** Chart depicts the timing of awakening, from the non-growing state, of cells that eventually survive ampicillin treatment. Approximately 69% of cells initiate growth during ampicillin treatment and the remaining ~31% initiate growth during the antibiotic washout recovery period.

**Figure S9**

**Supplementary Figure 9. Distribution of pre-exposure RpoS levels and lysis rates in slow-growth and fast-growth populations. a**, Immunoblot of RpoS levels in exponentially growing cells (~ 10^9^ cells) cultured in M9 and in M9+40X αMG. The values listed below represent normalized RpoS levels using RpoD levels as an endogenous control. **b,c** Comparison of RpoS-mCherry levels (**b**) and lysis rates (**c**) upon exposure to ampicillin (50 μg ml^-1^) in CFT073 cultured in M9 or M9+40X αMG. Each point represents individual UPEC cells, and red bars indicate means ± SD; asterisks (***) indicate significance (P<0.0001, Mann-Whitney test).


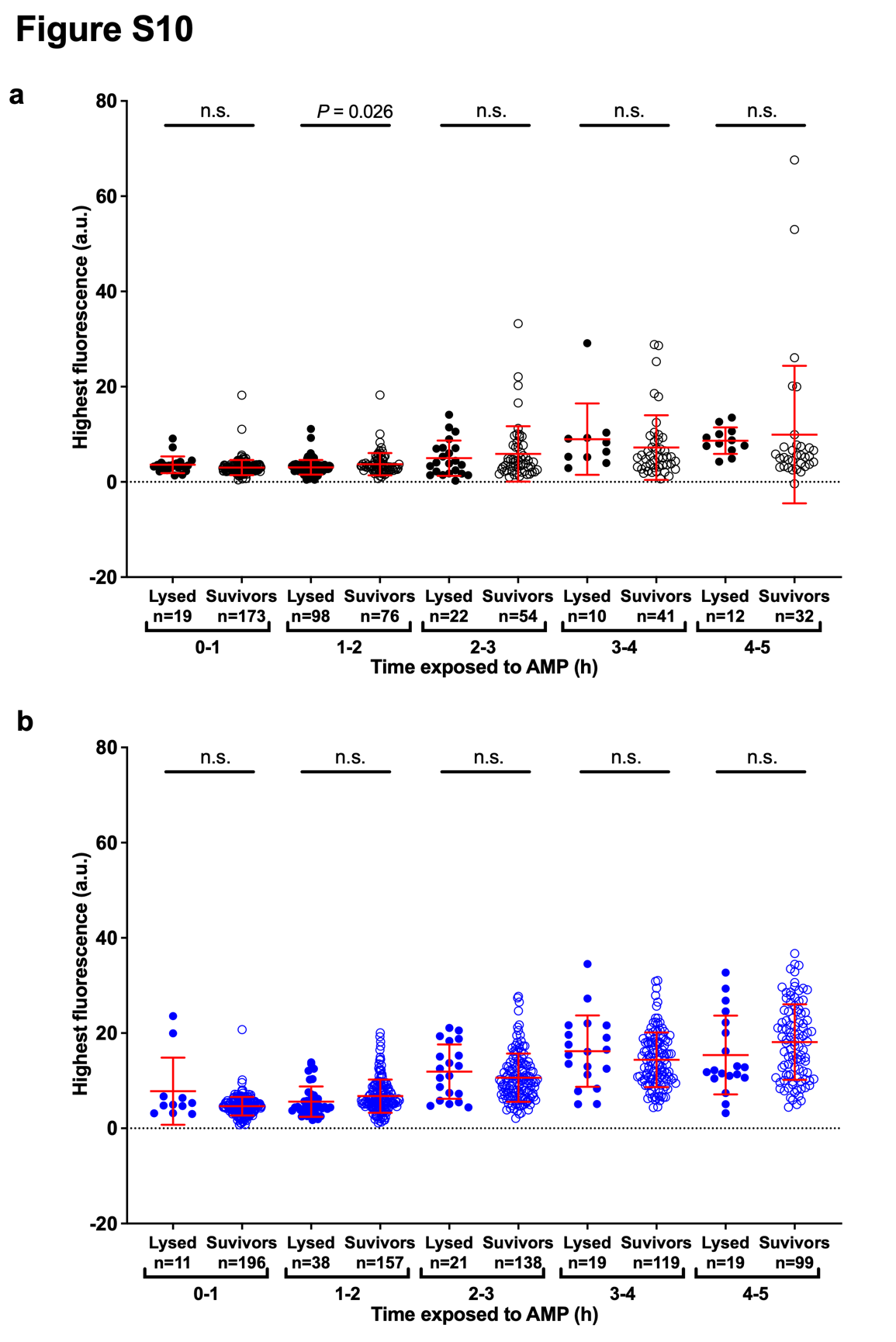


**Supplementary Figure 10. Transient higher expression of RpoS during ampicillin exposure is not involved in single-cell fates. a,b** Comparison of maximum RpoS-mCherry level in every hour of lysed cells to survived cells in M9 (**a**) and in M9+40X αMG (**b**).

**Figure S11**

**Supplementary Figure 11. RpoS is not required for UPEC persistence in M9 based media.** Wild type (WT) (black) or Δ*rpoS* (red) CFT073 were harvested from exponentially growing cultures (**a, c**) or from cultures in the stationary phase (**b, d**) and were exposed to ampicillin (50 μg ml^-1^) in M9 (**a, b**) or in M9+40X αMG (**c, d**). Aliquots were removed at the indicated time points, washed, and plated to determine survival. Data from two independent replicates have been plotted for each condition.

**Figure S12**

**
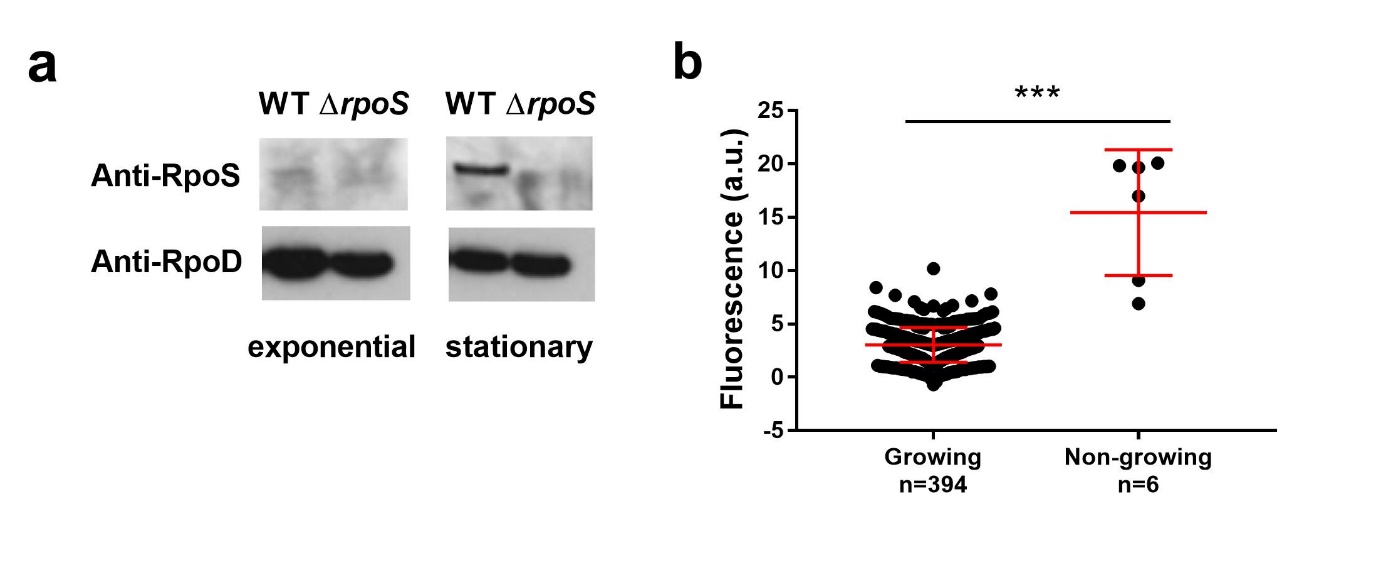
**

**Supplementary Figure 12. Non-growing bacteria have higher RpoS levels compared to growing cells. a)** Immunoblots showing RpoS levels in wild type (WT) or *ΔrpoS* strains of CFT073 harvested from exponential or stationary phase cultures. RpoD is the major housekeeping sigma factor and were used as controls. **b)** Comparison of RpoS-mCherry levels between growing cells and non-growing cells. Points show single-cell RpoS-mCherry levels and red bars indicate means±SD; asterisks (***) indicate significance (P<0.001, Mann-Whitney test).

**Figure S13**


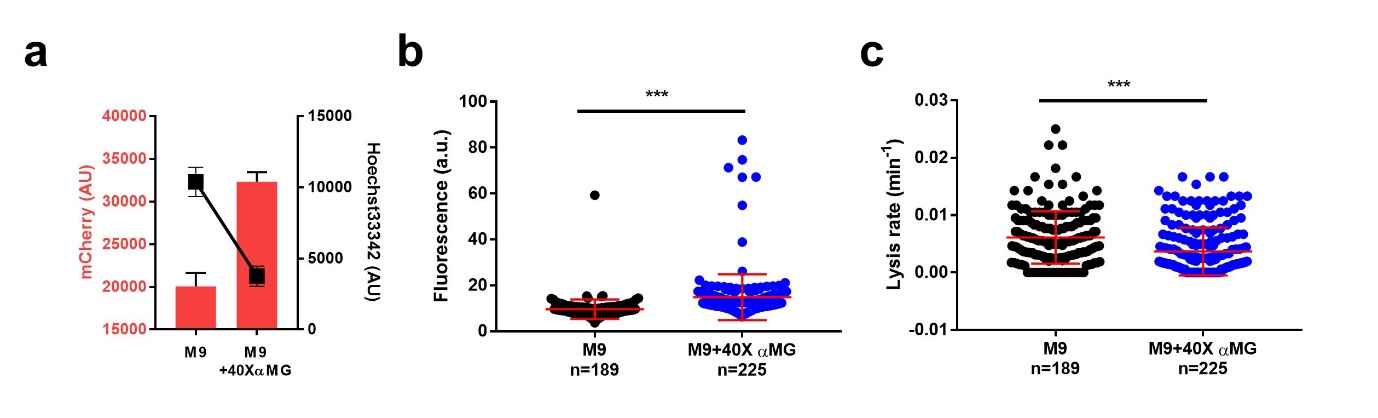


**Supplementary Figure 13. Distribution of pre-exposure efflux activities and death rates in slow-growth and fast-growth populations. a,** Comparison of mCherry fluorescence and Hoechst (H) 33342 accumulation levels between cells grown in M9 and in M9+40X αMG. Higher levels of *ptolC-mcherry* expression and lower levels of H33342 accumulation were seen in cells grown in M9+40X αMG. Lower levels of H33342 accumulation indicates higher efflux activity. Error bars represent means±SD (*n* = 3). **b,c** Comparison of single-cell *ptolC-mCherry* levels (**b**) and lysis rates (**c**) against to ampicillin (50 μg ml^-1^) between M9 and M9+40X αMG. Points show single-cell *ptolC-mCherry* levels or lysis rates, and red bars indicate means±SD; asterisks (***) indicate significance (P<0.0001, Mann-Whitney test).

**Figure S14**


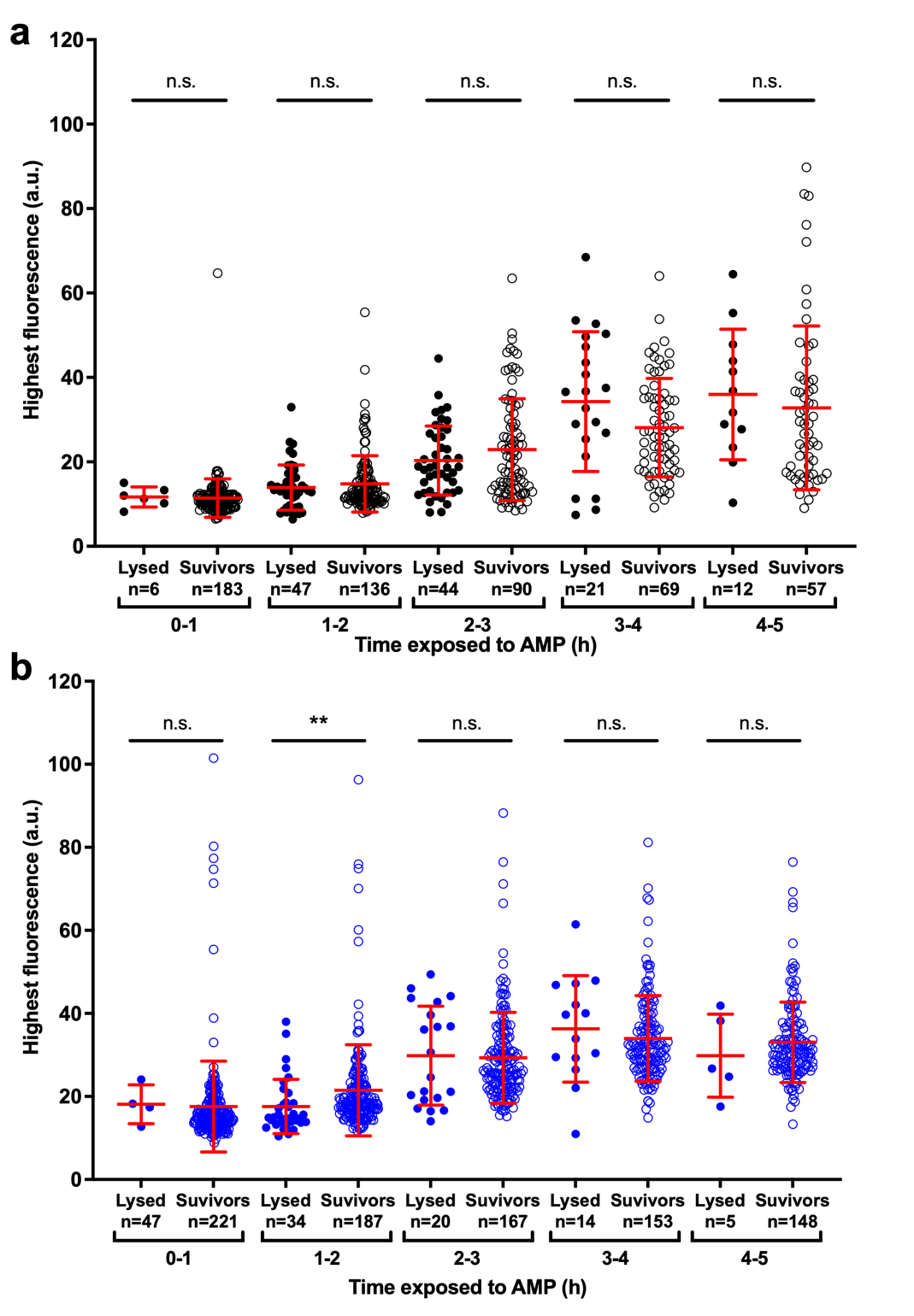


**Supplementary Figure 14. Transient higher efflux-activity during ampicillin exposure is not always involved in single-cell fates. a,b** Comparison of maximum *ptolc-mCherry* level in every hour of dead cells to survived cells in M9 (**a**) and in M9+40X αMG (**b**). asterisks (**) indicate significance (P<0.01, Mann-Whitney test.

**Supplementary Movie Legends**

**Supplementary Movie S1. Growing cells can survive during ampicillin treatment (first example).** UPEC strain CFT073 expressing YFP was cultured in a microfluidic device for ~ 13.2 hours under a constant flow of LB medium. Between 152-392 minutes (4 hours), 50 μg ml^-1^ ampicillin was added to the flow medium (“AMP” in the movie). Phase-contrast and fluorescence images were acquired at 4-minute intervals and merged. The arrow indicates a growing cell that survives and resumes growth after ampicillin washout. Scale bar, 10 μm.

**Supplementary Movie S2. Growing cells can survive during ampicillin treatment (second example).** UPEC strain CFT073 expressing YFP was cultured in a microfluidic device for ~ 15.1 hours under a constant flow of LB medium. Between 152-392 minutes (4 hours), 50 μg ml^-1^ ampicillin was added to the flow medium (“AMP” in the movie). Phase-contrast and fluorescence images were acquired at 4-minute intervals and merged. The arrow indicates a growing cell that survives and resumes growth after ampicillin washout. Scale bar, 10 μm.

**Supplementary Movie S3. Non-growing cells can survive during ampicillin treatment.** UPEC strain CFT073 expressing YFP was cultured in a microfluidic device for ~ 13.2 hours under a constant flow of LB medium. Between 148-388 minutes (4 hours), 50 μg ml^-1^ ampicillin was added to the flow medium (“AMP” in the movie). Phase-contrast and fluorescence images were acquired at 4-minute intervals and merged. The arrow indicates a non-growing cell that survives and resumes growth after ampicillin withdrawal. Scale bar, 10 μm.

**Supplementary Movie S4. Non-growing cells can be killed by ampicillin treatment.** UPEC strain CFT073 expressing YFP was cultured in a microfluidic device for ~ 7.2 hours under a constant flow of LB medium. Between 120-360 minutes (4 hours), 50 μg ml^-1^ ampicillin was added to the flow medium (“AMP” in the movie). Phase-contrast and fluorescence images were acquired at 4-minute intervals and merged. The arrow indicates a non-growing cell that dies (lyses) during ampicillin treatment. Scale bar, 10 μm.

**Supplementary Movie S5. The progeny of cells that survive during ampicillin treatment are not resistant to ampicillin.** UPEC strain CFT073 expressing YFP was cultured in a microfluidic device for ~ 9.3 hours under a constant flow of LB medium. Between 100-348 minutes (4 hours) and again between 500-560 minutes (1 hour), 50 μg ml^-1^ ampicillin was added to the flow medium (“AMP” in the movie). Phase-contrast and fluorescence images were acquired at 4-minute intervals and merged. The arrow indicates a growing cell that survives and regrows after ampicillin withdrawal (first exposure). The progeny of this survivor are not ampicillin resistant, as they are subsequently killed by exposure to the same dose of ampicillin (second exposure). Scale bar, 10 μm.

**Supplementary Movie S6. Growing cells can survive during ampicillin treatment in M9.** UPEC strain CFT073 expressing YFP was cultured in a microfluidic device for ~ 16.35 hours under a constant flow of M9 medium. Between 03:20-08:20 (5 hours), 50 μg ml^-1^ ampicillin was added to the flow medium (“AMP” in the movie). Phase-contrast and fluorescence images were acquired at 5-minute intervals and merged. The arrows indicate a growing cell that survives and resumes growth after ampicillin washout. Scale bar, 5 μm.

**Supplementary Movie S7. Growing cells can survive during ampicillin treatment in M9 + 40X aMG .** UPEC strain CFT073 expressing YFP was cultured in a microfluidic device for ~ 26 hours under a constant flow of M9 medium with αMG. Between 03:45-08:45 (5 hours), 50 μg ml^-1^ ampicillin was added to the flow medium (“AMP” in the movie). Phase-contrast and fluorescence images were acquired at 5-minute intervals and merged. The arrows indicate a growing cell that survives and resumes growth after ampicillin washout. Scale bar, 5 μm.

**Supplementary Movie S8. Non-growing cells survive during ampicillin treatment in M9.** UPEC strain CFT073 expressing YFP was cultured in a microfluidic device for ~ 19 hours under a constant flow of M9 medium. Between 03:40-08:40 (5 hours), 50 μg ml^-1^ ampicillin was added to the flow medium (“AMP” in the movie). Phase-contrast and fluorescence images were acquired at 10-minute intervals and merged. The arrow indicates a non-growing cell that survives and resumes growth after ampicillin washout. Scale bar, 5 μm.

**Supplementary Movie S9. Non-growing cells survive during ampicillin treatment in M9 + 40X aMG.** UPEC strain CFT073 expressing YFP was cultured in a microfluidic device for ~ 24 hours under a constant flow of M9 medium supplemented with αMG. Between 03:50-08:50 (5 hours), 50 μg ml^-1^ ampicillin was added to the flow medium (“AMP” in the movie). Phase-contrast and fluorescence images were acquired at 5-minute intervals and merged. The arrow indicates a non-growing cell that survives and resumes growth after ampicillin washout. Scale bar, 5 μm.

**Supplementary Movie S10. Non-growing cells can be killed by ampicillin treatment in M9.** UPEC strain CFT073 expressing YFP was cultured in a microfluidic device for ~ 19 hours under a constant flow of M9 medium. Between 03:05-08:05 (5 hours), 50 μg ml^-1^ ampicillin was added to the flow medium (“AMP” in the movie). Phase-contrast and fluorescence images were acquired at 5-minute intervals and merged. The arrow indicates a non-growing cell that dies (lyses) after ampicillin treatment. Scale bar, 5 μm.

**Supplementary Movie S11. Non-growing cells can be killed by ampicillin treatment in M9** **+ 40X aMG.** UPEC strain CFT073 expressing YFP was cultured in a microfluidic device for ~ 31.7hours under a constant flow of M9 medium supplemented with αMG. Between 03:50-08:50 (5 hours), 50 μg ml^-1^ ampicillin was added to the flow medium (“AMP” in the movie). Phase-contrast and fluorescence images were acquired at 10-minute intervals and merged. The arrow indicates a non-growing cell that dies (lyses) during ampicillin treatment. Scale bar, 5 μm.

**Supplementary Movie S12.** **Single-cell RpoS levels do not contribute to survival against ampicillin in M9.** UPEC strain CFT073 carrying *rpoS-mCherry* was cultured in a microfluidic device for ~ 15 hours under a constant flow of M9 medium. Between 03:05-08:05 (5 hours), 50 μg ml^-1^ ampicillin was added to the flow medium (“AMP” in the movie). Phase-contrast and fluorescence images were acquired at 10-minute intervals and merged. The arrow indicates a cell that survives and resumes growth after ampicillin treatment. Scale bar, 5 μm.

**Supplementary Movie S13. Single-cell RpoS levels do not contribute to survival against ampicillin in M9 + 40X aMG.** UPEC strain CFT073 carrying *rpoS-mCherry* was cultured in a microfluidic device for ~ 20 hours under a constant flow of M9 medium supplemented with αMG. Between 03:15-08:15 (5 hours), 50 μg ml^-1^ ampicillin was added to the flow medium (“AMP” in the movie). Phase-contrast and fluorescence images were acquired at 5-minute intervals and merged. The arrows indicate cells that survive and resume growth after ampicillin washout. Scale bar, 5 μm.

**Supplementary Movie S14. Single-cell TolC levels do not contribute to survival against ampicillin in M9.** UPEC strain CFT073 carrying *ptolC-mCherry* was cultured in a microfluidic device for ~ 14 hours under a constant flow of M9 medium. Between 03:00-08:00 (5 hours), 50 μg ml^-1^ ampicillin was added to the flow medium (“AMP” in the movie). Phase-contrast and fluorescence images were acquired at 5-minute intervals and merged. The arrows indicate cells that survive and resume growth after ampicillin washout. Scale bar, 5 μm.

**Supplementary Movie S15. Single-cell TolC levels do not contribute to survival against ampicillin in M9 + 40X aMG .** UPEC strain CFT073 carrying *ptolC-mCherry* was cultured in a microfluidic device for ~ 21 hours under a constant flow of M9 medium supplemented with αMG. Between 03:15-08:15 (5 hours), 50 μg ml^-1^ ampicillin was added to the flow medium (“AMP” in the movie). Phase-contrast and fluorescence images were acquired at 5-minute intervals and merged. The arrows indicate cells that survive and resume growth after ampicillin washout. Scale bar, 5 μm.

**Supplementary Movie S16. EHEC growing cells can survive during ampicillin treatment.** EHEC strain CIP 105917 expressing YFP was cultured in a microfluidic device for ~ 19 hours under a constant flow of LB medium. Between 294-555 minutes (4.35 hours), 50 μg ml^-1^ ampicillin was added to the flow medium (“AMP” in the movie). Phase-contrast and fluorescence images were acquired at 3-minute intervals and merged. The arrow indicates a growing cell that survives and resumes growth after ampicillin washout. Scale bar, 10 μm.
